# Multifaceted Suppression of Staphylococcal Virulence Phenotypes: *In Vitro* Study of a Cellular Status With Reduced Agr Activity and Impaired Biofilm

**DOI:** 10.1002/mbo3.70380

**Published:** 2026-09-13

**Authors:** Nghi Bao Nguyen, Yuri Ushijima, Raiku Sekiya, Kazuya Morikawa, Le Thuy Thi Nguyen

**Affiliations:** ^1^ Graduate School of Comprehensive Human Sciences University of Tsukuba Tsukuba Japan; ^2^ Center for Molecular Biomedicine University of Medicine and Pharmacy at HCMC HCM City Vietnam; ^3^ Division of Biomedical Science, Institute of Medicine University of Tsukuba Tsukuba Japan; ^4^ Biotechnology Centre of Ho Chi Minh City, Trung My Tay Ward HCM City Vietnam

**Keywords:** Agr system, anti‐biofilm, anti‐virulence, quorum sensing, *Staphylococcus aureus*

## Abstract

Anti‐quorum sensing (QS) therapy has been focused on to reduce the virulence of pathogenic bacteria. However, there is a risk that suppression of the staphylococcal QS Agr system might promote biofilm formation. In this study, we found that both Agr activity and the biofilm of *Staphylococcus aureus* can be reduced by a sappanwood extract (SWe). The impaired biofilm in the presence of SWe exhibited reduced extracellular DNA (eDNA) and increased extracellular polysaccharides. SWe also reduced pigmentation. The RNA‐seq analysis showed that SWe suppressed multiple key virulence regulators, including the Agr and SaeRS systems. SWe downregulated cell‐wall‐associated proteins and several Agr‐regulated virulence factors such as hemolysins and phenol‐soluble modulins (PSMs). The present study did not intend the clinical application of the SWe but exemplified that it is possible to suppress multiple virulence phenotypes simultaneously *in vitro*, suggesting a new therapeutic concept to combat *S. aureus* infection.

## Graphical abstract

Finding of *in vitro S. aureus* status with reduced Agr activity and impaired biofilm.

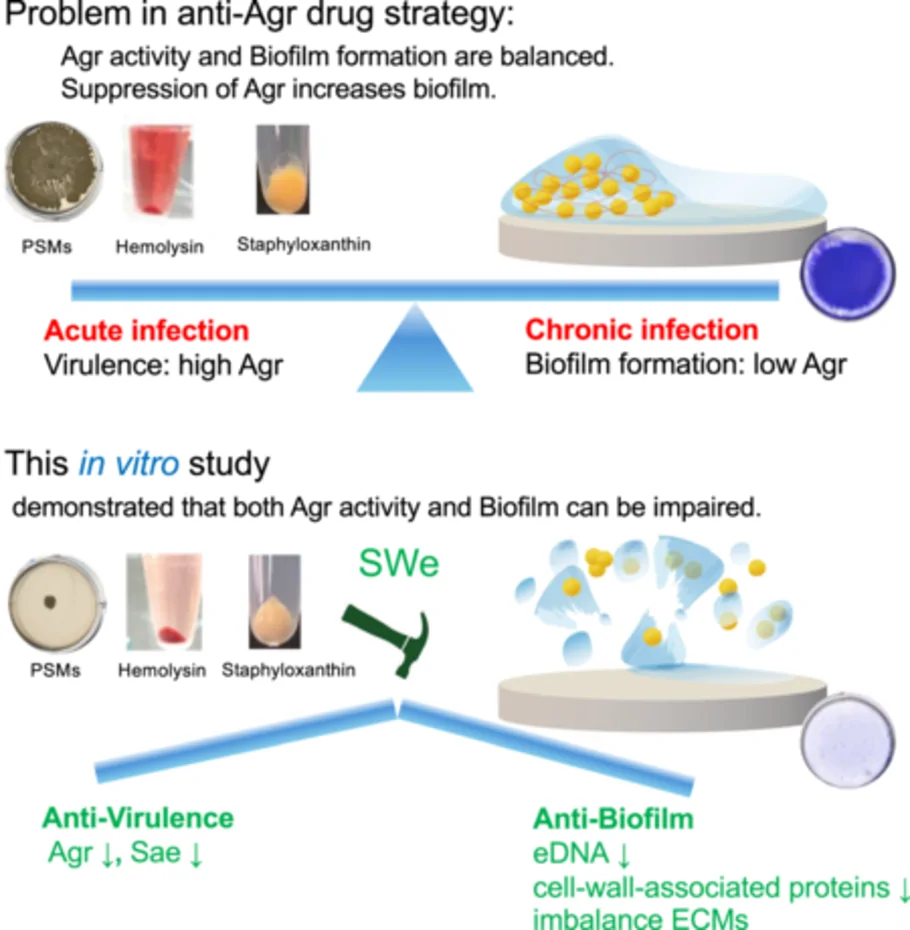

## 1 Introduction

*Staphylococcus aureus* is a Gram‐positive bacterium that asymptomatically inhabits the nasal cavities and skin surfaces of humans and animals, but it can cause diverse diseases (Humphreys 2012). Methicillin‐resistant *S. aureus* (MRSA) is notorious for its resistance to β‐lactams and poses a burden on a global scale (Guo et al. 2020; Murray et al. 2022).

In addition to the antibiotic‐resistance genes, *S. aureus* also forms the biofilm that protects microbes from host immune attacks and many antibiotics, making it difficult to eradicate infections (Jacqueline and Caillon 2014) (Paharik and Horswill 2016). They are surrounded by a matrix of extracellular polysaccharides, extracellular and cell surface‐associated proteins/adhesins, and extracellular DNA (eDNA) (Moormeier and Bayles 2017). The main extracellular polysaccharide in *S. aureus* biofilm is poly‐N‐acetylglucosamine (PNAG), also known as polysaccharide intercellular adhesin (PIA), which is synthesized by the enzymes encoded by *ica*ADBC operon (Nguyen et al. 2020). Although most *S. aureus* strains possess the *ica* genes, a subset does not depend on the PIA production to form biofilms, and the biofilm of *S. aureus* is divided into two groups: PIA‐dependent biofilm and PIA‐independent biofilm (McCarthy et al. 2015). PIA‐dependent biofilm is mainly found in methicillin‐sensitive *S. aureus* (MSSA), while MRSA prominently produces PIA‐independent biofilm (Nguyen et al. 2020). The biofilm formation of *S. aureus* has been illustrated through the five development stages: attachment, multiplication, exodus, maturation, and dispersal (Moormeier and Bayles 2017; Paharik and Horswill 2016) (Fig S1).

The Agr system is the master regulator of virulence in *S. aureus* and is activated through a quorum‐sensing/diffusion‐sensing mechanism (Fig S2) (Le and Otto 2015). In general, the Agr system turns on the expression of a series of secretory toxins/enzymes and turns off the expression of factors for surface attachment and biofilm formation. The Agr system is critical in invasive infection, and some inhibitors that target the Agr system are successful in pre‐clinical trials (Ford et al. 2020; Tuchscherr and Otto 2023; Vinodhini and Kavitha 2024). On the other hand, Agr activity is thought to hinder chronic survival in the human body, and a considerable part of the clinical isolates has no Agr activity due to mutations (Gor et al. 2019). These indicate the difficulty in dealing with the staphylococcal infection strategies by inhibiting the Agr system alone.

A series of studies indicated that the Agr system negatively regulates biofilm (Paharik and Horswill 2016), and the suppression of Agr could lead to the promotion of biofilm. Although the increase in biofilm in the *agr*‐deficient strains could depend on the limited conditions *in vitro*, and may not correlate with the *in vivo* bacterial behavior (Jordan et al. 2022), the repeatedly reported negative effect of Agr on biofilm has discouraged the idea of the application of the Agr inhibitors to the treatment, e.g. (Vuong et al. 2000).

The role of Agr in biofilm development involves multiple direct/indirect Agr regulon factors (Fig S1, S2). At the early stage of biofilm formation, where the Agr is not activated, cell wall‐associated proteins are expressed to facilitate cell attachment and local multiplication. In the dispersal stage, the Agr system plays a role in inducing exoproteins such as proteases and PSMs. It is also reported that the inactivation of Agr disables the release of cells from the *in vivo* biofilm in the mouse model (Nishitani et al. 2015). Proteases contribute to the dispersal of biofilm (Boles and Horswill 2008). PSMs play multiple roles depending on their forms: soluble form, amyloid form, or complex with DNA, and the absence of PSMs could decrease biofilms (Howard et al. 2023) (Grando et al. 2022) (Schwartz et al. 2012), or compact and thicken biofilm (Periasamy et al. 2012).

Through our ongoing efforts to find plant extracts applicable to the treatment of bacterial infections, the dichloromethane (DCM) extract of *Biancaea sappan* (formerly *Caesalpinia sappan* (Gagnon et al. 2016)) was found to have unexpected *in vitro* characteristics: it suppressed both virulence phenotypes and biofilm in *S. aureus*. *Biancaea sappan*, commonly known as sappanwood or Indian redwood, is a tropical tree native to Southeast Asia. The sappanwood tree has been used in traditional oral medicine to treat conditions such as inflammation and pain (Vij et al. 2023). In this study, we aimed to clarify the *in vitro* cellular status of *S. aureus*, where the Agr activity and biofilm are both impaired in response to the sappanwood extract.

## 2 Results

### 2.1 SWe Impairs the Biofilm

**Figure 1:**
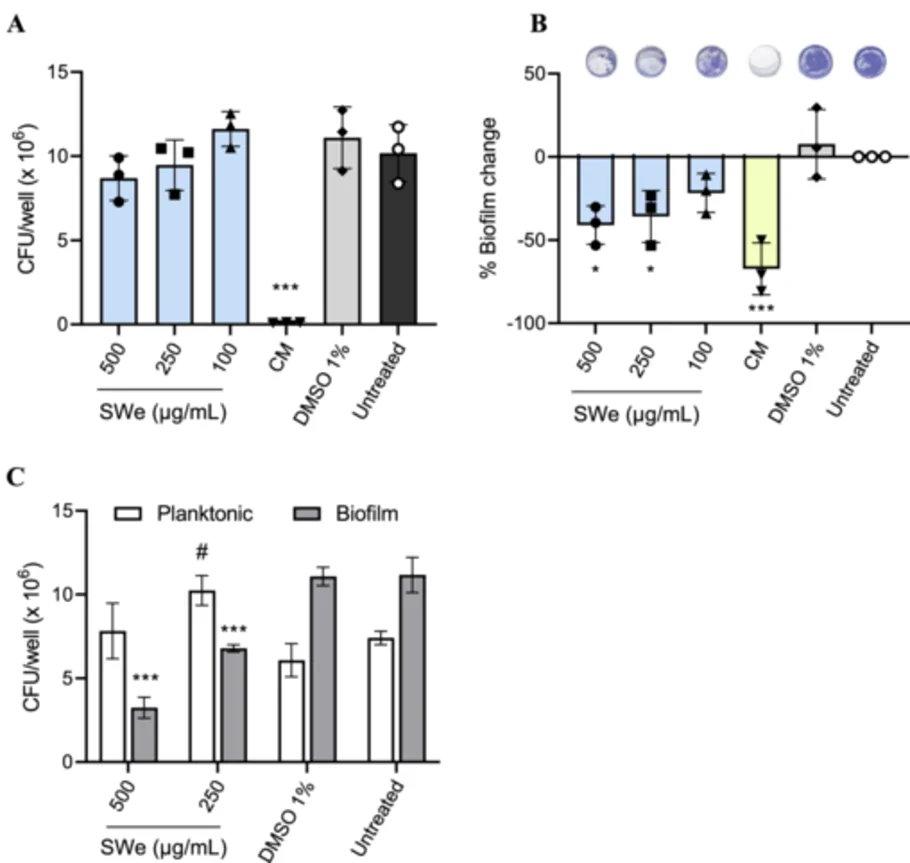
The antibiofilm activity of SWe on SH1000 after 24 h of treatment. (A) Cell viability in total biomass (biofilm and planktonic cells) relative to untreated sample. The viable cells were counted as CFU. (B) Biofilm was quantified using crystal violet, and the values of percent change relative to the untreated group are shown. Representative images of stained biofilms are shown above the graph. (C) The viable cells were counted as CFU separately in planktonic (blank bars) and biofilm (filled bars) status. All data represent the mean values of 3 independent experiments; error bars indicate the SD. *p ≤ 0.05, **p ≤ 0.001, ***p ≤ 0.0001 compared to the untreated group, #*p* ≤ 0.05 planktonic cells compared with those in the untreated group. One‐way ANOVA for (A, B) and Two‐way ANOVA for (C) & Dunnett's multiple comparison test with the untreated group. CM: 62.5 μg/mL chloramphenicol.

*S. aureus* SH1000 was selected as a representative strain to study the effect of the sappanwood extract (SWe). SH1000 is the derivative of the historically well‐studied laboratory strain 8325‐4 (Horsburgh et al. 2002). It is Agr positive and makes PIA‐dependent biofilm (Horsburgh et al. 2002; Izano et al. 2008). The minimal inhibitory concentration (MIC) value of SWe on SH1000 was ~500 µg/mL in MH broth and TSB (Table S1). In the biofilm assay conditions, where we inoculated a higher quantity of cells (10^6^ CFU per well) than in the MIC assay (10^5^ CFU per well), the CFU did not significantly change by 250 ug/mL SWe (95% compared with untreated control) (Figure 1A). Even at 500 µg/mL SWe, CFU was 80% of the untreated control, while the addition of MIC of chloramphenicol (62.5 µg/mL) reduced the CFU to an undetectable level. Thus, SWe is not very effective in its antibacterial activity in the biofilm assay conditions employed. Nevertheless, the biofilm of SH1000 was reduced by 25%, 35%, and 40% by 100, 250, and 500 µg/mL SWe (Figure 1B). Here, the cells were easy to disperse in the presence of SWe and were unable to firmly establish a biofilm (**Supplementary Movie**). SWe did not affect the initial attachment of cells to the 96‐well polystyrene plate (Figure S3). The anti‐biofilm effect of 250 µg/mL SWe was also observed in another medium, BHI + 3% NaCl, that is often employed for the analysis of biofilms (Chiba et al. 2022; O'Neill et al. 2007) (Figure S4). Thus, it is likely that SWe has anti‐biofilm activity. Here, it must be noted that there are many reports where the growth inhibition was mistakenly regarded as the anti‐biofilm activity. We therefore measured the CFU values of both biofilm and planktonic (washable) parts. If SWe has *bona fide* anti‐biofilm effect, it would reduce biofilm CFU without reducing the CFU in the planktonic part. As expected, we could observe that the 250 and 500 µg/mL SWe did not reduce the CFU value of planktonic cells while reducing that of biofilm (Figure 1C). The CFU in the planktonic part was increased by SWe, but it is not always significant in different lots (data not shown). In summary, SWe interferes with SH1000 cells in biofilm development or maintenance without severe antimicrobial activity.

### 2.2 SWe Reduces eDNA in Biofilm

**Figure 2:**
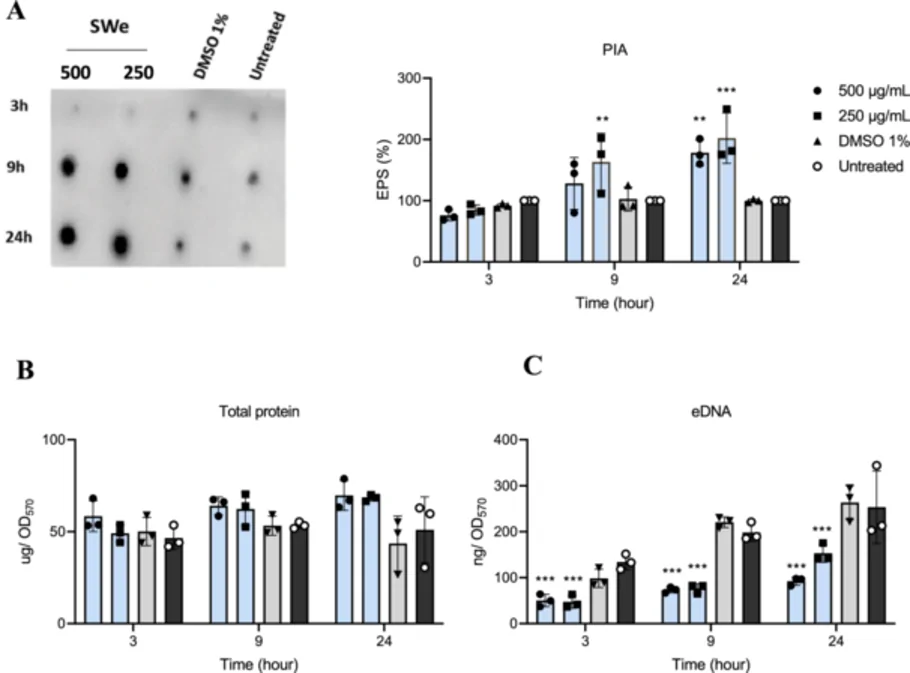
Quantification of ECM components in SH1000 biofilm treated with SWe at 3, 9, and 24 h. (A) PIA was detected by WGA staining (left). The signal intensity was quantified, and normalized by biofilm biomass (OD_570_), and the relative values compared with the untreated group are shown (right). (B) Extracellular protein (µg) normalized by OD_570_. (C) eDNA (ng) normalized by OD_570_. All data represent the average from 3 independent experiments; error bars indicate the SD. Two‐way ANOVA & Dunnett's multiple comparison test with the untreated group, **p ≤ 0.001, ***p ≤ *0.0001*.

Extracellular polysaccharides (EPS, also known as PIA), extracellular protein, and eDNA are the major constituents in *S. aureus* biofilm. These were quantified at different time points (3, 9, and 24 h) and were normalized by O.D._600_. Unexpectedly, PIA in the SWe‐treated group significantly increased at 9 and 24 h (Figure 2A). In addition, assays using the poly‐β−1,6‐N‐acetylglucosamine (PNAG)‐specific enzyme Dispersin B and its derivative indicated that SWe increased PNAG (Figure S5). The quantity of extracellular protein was not changed by SWe (Figure 2B). Instead, we observed a significant reduction in the eDNA amount in the SWe‐supplemented biofilms throughout the tested time points (Figure 2C). The source of the eDNA is the genomic DNA released by the autolysis, and indeed, we found SWe reduces the autolysis (Figure S6). In addition, the biofilm degradation assay (Figure S7) also suggested the qualitative change in the biofilm.

### 2.3 SWe Suppresses the Agr Activity

**Figure 3:**
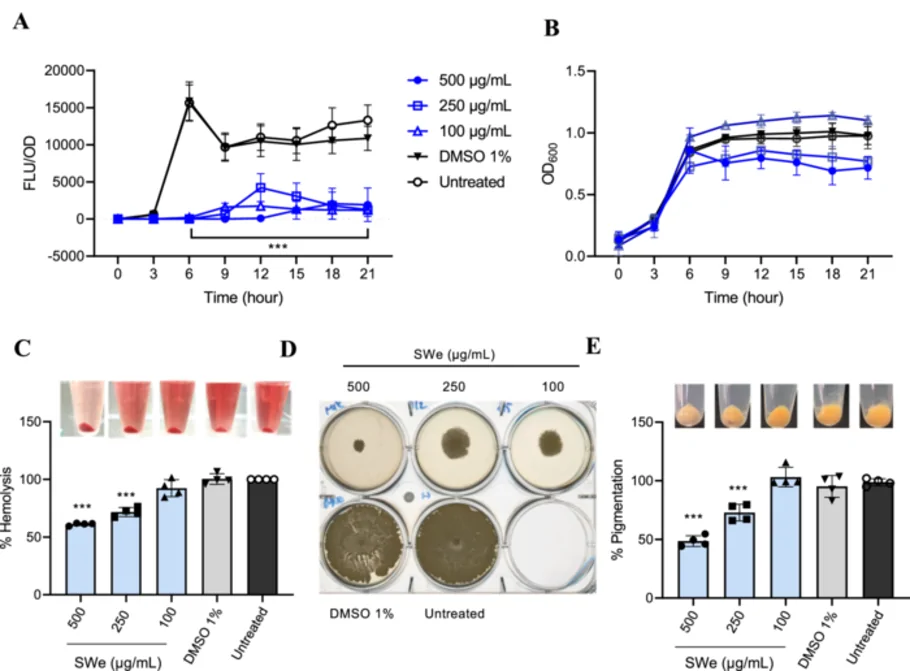
Inhibition of Agr system and virulence factors by SWe in SH1000 (18 h). (A) The reporter signal was normalized by an OD_600_ value of SH1000 P3*venus*. (B) Growth curves of the reporter strain at different treatment concentrations. (C) Hemolysis activity of SH1000 after treating with SWe. (D) Colony spreading of SH1000. SWe was supplemented in the agar medium. (E) Pigmentation of SH1000. Photos of cell pellets after centrifugation are shown above the graph. All data represent the average of 3 or 4 independent experiments; error bars indicate the SD. Statistical significance (**p ≤ 0.001, ***p ≤ *0.0001*) was observed for SWe treated groups (500, 250 and 100 μg/mL). One‐way ANOVA for (C, E) and Two‐way ANOVA for (A, B) & Dunnett's multiple comparison test with the untreated group.

The effect of SWe on the Agr activity was tested using the reporter strain SH1000 P3*venus* in planktonic conditions (Figure 3A, Figure S8). The reporter plasmid expresses the fluorescence gene under the control of the *agr* P3 promoter. The control without SWe induced the fluorescence signal around 6 h and then sustained a high level. On the other hand, the signal in the SWe‐treated groups was suppressed. The suppression of fluorescence in the treated groups was not due to the delay or reduction of growth (Figure 3B). As observed under microscopy, the number of fluorescent cells was higher in the untreated group, and SWe reduced the fluorescent cells (Figure S8).

The effect of SWe on the SH1000 virulence phenotypes *in vitro* was further tested in terms of hemolysis and colony spreading, both are regulated by the Agr system (Figure S2). The hemolytic activity of SH1000 was decreased by the addition of SWe to the growth medium, and the effect was dose‐dependent (Figure 3C). Similarly, the spreading of SH1000 on soft agar, to which phenol soluble modulins (PSMs) mainly contribute (Kizaki et al. 2016), was suppressed by SWe (Figure 3D).

We also noticed that SWe reduces the yellow pigment production of SH1000 (Figure 3E). The carotenoids, such as staphyloxanthin are virulence factors that support *S. aureus* survival under oxidative stress (Xue et al. 2019). The pigment in the treated SH1000 was significantly dropped by 50% and 35% at 500 and 250 µg/mL SWe, respectively. The pigmentation of *S. aureus* depends on the alternative sigma factor SigB (Kullik et al. 1998) and is not directly regulated by the Agr system, but some evidence shows the deletion of Agr reduces pigmentation (Marroquin et al. 2019; Tan et al. 2022).

### 2.4 Cellular Status in the Presence of SWe Is Revealed by RNA Sequencing

So far, we have demonstrated that SWe can make *S. aureus* SH1000 into an Agr‐suppressed and biofilm‐impaired status. The transcriptome was analyzed by RNA sequencing (RNA‐seq) to gain additional insight into this interesting cellular status. Cells were statically grown as in biofilm assays, and all cells in each well (as a mixture of planktonic and biofilm cells) were submitted to the analysis. There were about 100–150 genes expressed differently (log_2_FC > 1 and *p*‐value < 0.05) in 250 µg/mL SWe conditions compared with controls at 3, 9, and 24 h (Figure S9, Table S2–S4). Fifty‐three downregulated genes and 33 upregulated genes were shared across at least two time points (Tables 1, 2).

**Table 1:** Downregulated genes by SWe at two or more time points in 3, 9, and 24 h (*p* < 0.05).

Gene	Function	Gene expression		
		3 h	9 h	24 h
efb	complement convertase inhibitor Efb	−7.91	−3.07	−6.05
ecb	complement convertase inhibitor Ecb	−7.27	−2.73	−5.08
scb	complement inhibitor SCIN‐B	−6.55	−1.99	−4.99
sbi	immunoglobulin‐binding protein Sbi	−1.97	−2.29	−5.01
eap	extracellular adherence protein Eap/Map	−4.13	−3.75	−2.47
saeP	DM13 domain‐containing protein	−3.92	−2.43	−4.29
saeQ	DoxX family protein	−1.88	−2.51	−3.58
sarY	SarA family transcriptional regulator	−3.33	−1.27	−2.49
hlgC	bi‐component gamma‐hemolysin HlgCB subunit C	−1.77	−3.26	−3.25
hyl	alpha‐hemolysin	−3.17	−2.25	−5.48
SAURSH1000_RS07670	SAS049 family protein	−5.20	−1.16	−1.80
SAURSH1000_RS05335	beta‐class phenol‐soluble modulin	−4.80	−1.09	−2.95
SAURSH1000_RS12605	hypothetical protein	−5.57	−3.42	—
SAURSH1000_RS07170	exodeoxyribonuclease VII small subunit	−5.31	−5.68	—
yhbY	ribosome assembly RNA‐binding protein YhbY	−5.31	−2.78	—
SAURSH1000_RS12025	hypothetical protein	−5.00	−2.33	—
SAURSH1000_RS03245	hypothetical protein	−4.40	−1.26	—
SAURSH1000_RS07675	CsbD family protein	−4.38	−1.20	—
SAURSH1000_RS03815	DUF5067 domain‐containing protein	−3.66	−1.39	—
SAURSH1000_RS00845	isoprenylcysteine carboxyl methyltransferase family protein	−3.65	−2.18	—
SAURSH1000_RS08250	hypothetical protein	−3.15	−1.39	—
SAURSH1000_RS00240	phosphatidylinositol‐specific phospholipase C	−2.81	−1.95	—
SAURSH1000_RS06945	hypothetical protein	−2.76	−2.26	—
SAURSH1000_RS06090	polymorphic toxin type 50 domain‐containing protein	−2.56	−1.94	—
SAURSH1000_RS04915	YkyA family protein	−2.30	−1.33	—
SAURSH1000_RS03810	hypothetical protein	−2.01	−1.76	—
SAURSH1000_RS03270	hypothetical protein	−1.87	−1.16	—
SAURSH1000_RS02080	hypothetical protein	—	−2.66	−5.45
hlgB	bi‐component gamma‐hemolysin HlgAB/HlgCB subunit B	—	−2.60	−2.24
hlgA	bi‐component gamma‐hemolysin HlgAB subunit A	—	−1.89	−4.07
saeR	response regulator transcription factor SaeR	—	−1.87	−2.12
saeS	two‐component system sensor histidine kinase SaeS	—	−1.53	−2.14
cntA	staphylopine‐dependent metal ABC transporter substrate‐binding protein CntA	—	−1.41	−3.69
SAURSH1000_RS08665	DUF4888 domain‐containing protein	—	−1.64	−4.77
SAURSH1000_RS02680	long‐chain fatty acid‐‐CoA ligase	—	−2.55	−3.33
SAURSH1000_RS01930	phenol‐soluble modulin PSM‐alpha‐4	−6.83	—	−3.37
SAURSH1000_RS01935	phenol‐soluble modulin PSM‐alpha‐2	−6.75	—	−3.53
isdC	heme uptake protein IsdC	−6.29	—	−2.29
SAURSH1000_RS13275	MAP domain‐containing protein	−5.31	—	−2.65
SAURSH1000_RS05340	beta‐class phenol‐soluble modulin	−5.24	—	−4.24
sarR	HTH‐type transcriptional regulator SarR	−5.12	—	−1.84
SAURSH1000_RS13285	delta‐lysin family phenol‐soluble modulin	−4.56	—	−2.33
sph	orotate phosphoribosyltransferase	−3.80	—	−2.39
sdrC	MSCRAMM family adhesin SdrC	−3.08	—	−2.11
SAURSH1000_RS08740	tRNA‐Ser	−2.91	—	−3.50
sdrD	MSCRAMM family adhesin SdrD	−2.70	—	−2.01
SAURSH1000_RS03975	CsbD family protein	−2.43	—	‐2.35
SAURSH1000_RS05245	hypothetical protein	‐2.17	—	‐3.24
SAURSH1000_RS03135	hypothetical protein	‐2.10	—	‐1.75
SAURSH1000_RS06035	hypothetical protein	‐1.84	—	‐2.58
SAURSH1000_RS01685	GlsB/YeaQ/YmgE family stress response membrane protein	‐1.76	—	‐2.03
SAURSH1000_RS01675	PepSY domain‐containing protein	‐1.69	—	‐2.52
SAURSH1000_RS03900	histidine phosphatase family protein	‐1.62	—	‐1.85

**Table 2:** Upregulated genes by SWe at two or more time points in 3, 9, and 24 h (*p* < 0.05).

Gene	Function	Log2FC		
		3 h	9 h	24 h
SAURSH1000_RS06115	ABC transporter ATP‐binding protein	7.40	4.85	2.39
farE	fatty acid efflux MMPL transporter FarE	7.23	4.47	5.88
SAURSH1000_RS06120	ABC transporter permease	4.80	7.43	2.39
SAURSH1000_RS12500	hypothetical protein	4.80	4.26	1.68
SAURSH1000_RS12505	DUF896 domain‐containing protein	3.93	4.53	1.68
ptsG	glucose‐specific PTS transporter subunit IIBC	2.89	2.48	2.40
SAURSH1000_RS06125	sensor histidine kinase	2.69	3.23	2.39
ureB	urease subunit beta	2.52	1.91	1.68
ureA	urease subunit gamma	2.33	2.09	1.68
ureC	urease subunit alpha	1.75	2.19	1.68
ureD	urease accessory protein UreD	1.73	2.21	1.68
ureG	urease accessory protein UreG	1.71	2.05	1.68
SAURSH1000_RS06130	response regulator	2.39	3.91	2.39
sceD	lytic transglycosylase SceD	2.36	1.09	1.68
SAURSH1000_RS01980	sodium‐dependent transporter	3.19	1.20	—
SAURSH1000_RS11325	HlyD family efflux transporter periplasmic adaptor subunit	3.14	1.50	—
SAURSH1000_RS00800	FMN‐dependent NADH‐azoreductase	2.28	1.34	—
SAURSH1000_RS02965	tyrosine‐type recombinase/integrase	2.11	1.46	—
SAURSH1000_RS06745	PepSY domain‐containing protein	2.01	1.31	—
SAURSH1000_RS13115	SMP‐30/gluconolactonase/LRE family protei	1.69	1.03	—
SAURSH1000_RS12525	TetR/AcrR family transcriptional regulator	1.68	1.39	—
SAURSH1000_RS11460	sucrose‐specific PTS transporter subunit IIBC	1.51	1.23	—
SAURSH1000_RS11320	DHA2 family efflux MFS transporter permease subunit	3.36	—	2.18
SAURSH1000_RS12275	PTS fructose transporter subunit IIC	2.32	—	1.72
sdaAA	l‐serine ammonia‐lyase, iron‐sulfur‐dependent, subunit alpha	2.15	—	2.05
ureE	urease accessory protein UreE	—	1.77	4.13
ureF	urease accessory protein UreF	—	1.61	4.06
SAURSH1000_RS12330	sterile alpha motif‐like domain‐containing protein	—	1.59	2.77
rpmB	50S ribosomal protein L28	—	1.35	2.19
SAURSH1000_RS06900	HU family DNA‐binding protein	—	1.29	2.30
fadA	thiolase family protein	—	1.67	4.50
fadB	3‐hydroxyacyl‐CoA dehydrogenase/enoyl‐CoA hydratase family protein	—	1.26	4.22
SAURSH1000_RS11850	APC family permease	—	1.06	1.68

**Figure 4:**
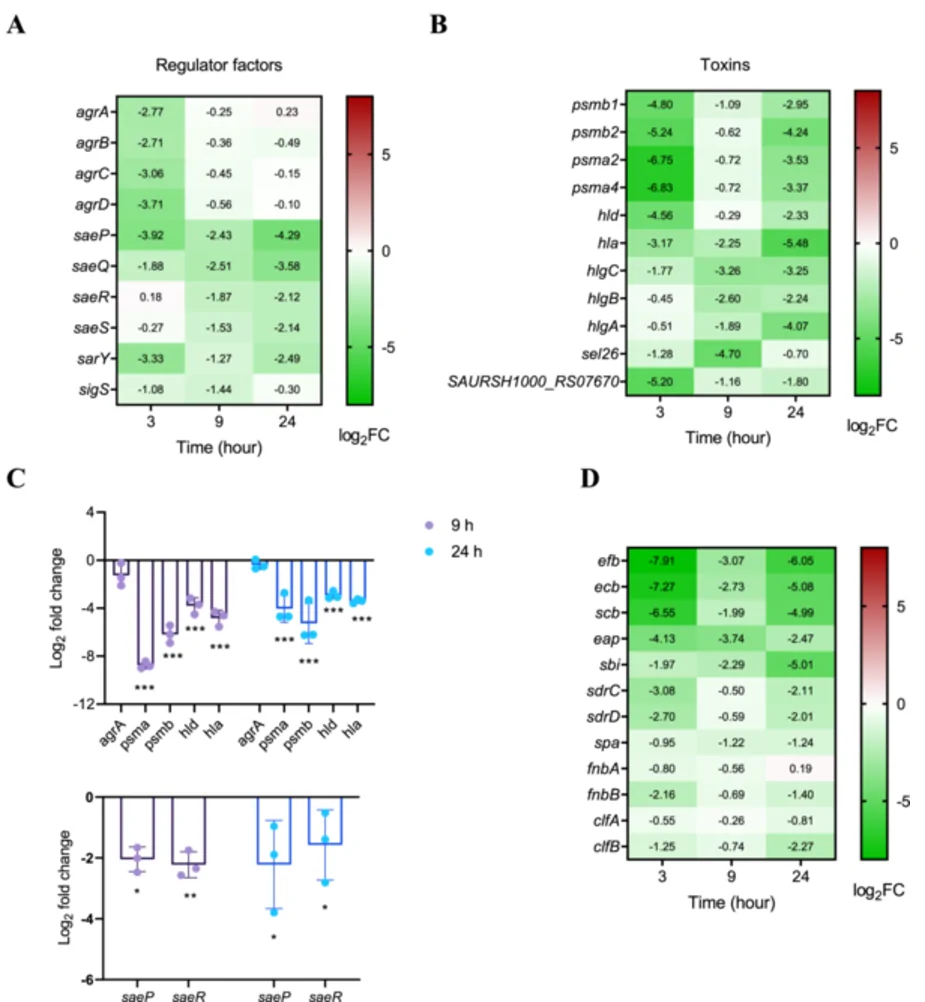
Differentially Expressed Genes (DEGs) in the SWe‐treated cells: adhesins, exotoxins, and regulators. Log_2_ (Fold Change) values relative to untreated control at the same time point are shown. DEGs were grouped into regulator factors (A), exotoxins (B), and adhesins (D). (C) qPCR confirmation of the expression of selected genes at 9 h and 24 h. Error bars represent the SD; *p ≤ 0.05, **p ≤ 0.001, ***p ≤ *0.0001*. Fold change in expression levels was calculated by the 2^−ΔΔCT^ method. Statistical analysis was performed by unpaired t‐test for the log_2_(fold change expression) values.

The expression of *agrBDCA* operon was decreased at 3 h in RNA‐seq data (Figure 4A). Consistently, the Agr‐regulated virulence genes (*psma, psmb, hld, hla*) were suppressed even in the 24 h treatment (Figure 4B) and were confirmed by qPCR (Figure 4C). The expression of another quorum‐sensing regulator gene, *luxS*, which can affect biofilm (Chen et al. 2025), was not significantly changed by SWe (Figure SS10).

The RNA‐seq analysis alone did not fully explain the increased PIA production (Figure 2A) or the decreases in eDNA levels (Figure 2B) and autolytic activity (Figure S6). In terms of PIA synthesis genes (*ica* operon), the expression was not increased in some genes and time points (Figure S11). Instead, we observed that the genes for the capsule biosynthesis pathway, which shares UDP‐GlcNac as the starting material with the PIA biosynthesis pathway, were downregulated under treatment of SWe at 3 h (*capA* through *capP*) (Figure S11). RT‐PCR analysis confirmed the slight reduction of *capAB* at 24 h (Figure S10).

Many genes encoding adhesion proteins were downregulated in the SWe‐treated sample (Figure 4D). Indeed, we observed that SWe‐treated cells are impaired in their fibronectin binding capability (Figure S12). This is one of the important findings in this study because adhesin proteins are known to be negatively regulated by the Agr system (Le and Otto 2015). The evasion proteins extracellular fibrinogen‐binding protein (Efb), extracellular complement‐binding protein (Ecb), staphylococcal complement inhibitor B (Scb), staphylococcal binder of IgG (Sbi), and extracellular adherence protein (Eap) have been implicated in the *S. aureus* biofilm, possibly through positive charge–mediated interactions with eDNA and the cell wall (Dengler et al. 2015; Graf et al. 2019; Kavanaugh et al. 2019). Together with the low amount of eDNA in ECM (Figure 2C), the decrease in these adhesion proteins would affect the biofilm of *S. aureus* under treatment of SWe. Moreover, other MSCRAMMs involved in biofilm formation (FnBPA, FnnBPB, SdrC, SdrD, ClfA, ClfB, and SpA) tended to be lower in the SWe‐treated sample. It is known that Efb, Sbi, Eap, FnBPA, FnBPB, and complement inhibitors are under the control of the two‐component system SaeRS (Liu et al. 2016). Consistently, the expression of the SaePQRS was reduced by SWe (Figure 4C,D). SaeP is the bacterial cell surface protein required for the stability of biofilm by interaction with eDNA in ECM (Kavanaugh et al. 2019). Thus, the expression of some types of eDNA‐binding proteins in ECM was reduced in SWe conditions, likely through the downregulation of the SaePQRS systems.

SWe also affected the expression of genes related to some metabolic pathways (Fig S13), and the intracellular level of reactive oxygen species (ROSs) (Figure S14), but their contribution to suppress virulence phenotypes and biofilm is not known.

In summary, our transcriptome analysis confirmed the inhibitory effect of SWe on the Agr system. It also found the suppression of adhesin genes and the Sae system. Suppression of these virulence regulators is consistent with the SWe's effect on *in vitro* virulence phenotypes and biofilm. Regarding the mechanism by which this ‘avirulent’ transcriptome status is achieved, further extensive studies are required, including the identification of the active compounds. In the present study, we can only mention that the SWe's major anti‐biofilm effect is independent of the Agr system; SWe suppressed the biofilm in the absence of the Agr system (Figure S15). SWe also reduced biofilm of SA113 (*rsbU* has a mutation), suggesting that RsbU (SigB positive regulator) is not essential for the SWe's anti‐biofilm effect (Figure S16), though it may partly contribute.

### 2.5 Effect of SWe on Different Types of *S. aureus* Strains

**Figure 5:**
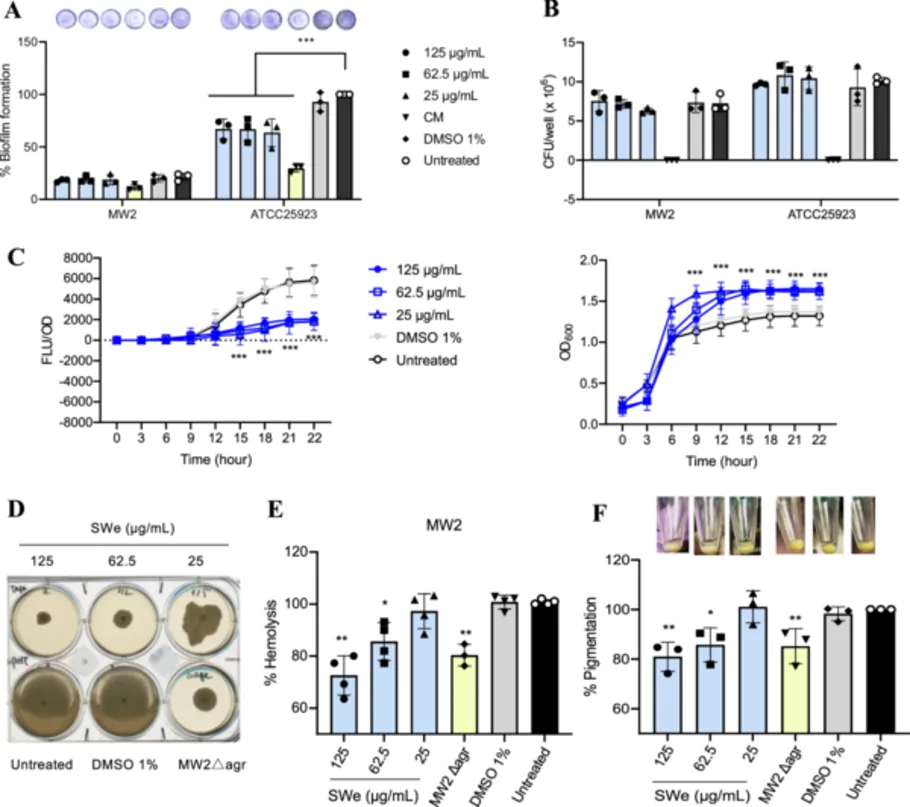
Effect of SWe on biofilm formation and virulence expression of MW2 and ATCC25923. Biofilm formation (A) and viability (B) of MW2 and ATCC25923 in the presence of SWe. The relative percentages to the untreated ATCC25923 biofilm is shown. (C) RNA‐III reporter assay to test the anti‐Agr activity of SWe on MW2. MW2 virulence phenotypes were also tested: (D) colony spreading, (E) hemolysis, and (F) pigmentation. All data represent the mean values of three independent experiments; error bars indicate the SD. Statistical significance (*p ≤ 0.05, **p ≤ 0.001, ***p ≤ 0.0001) was observed for SWe treated groups (125, 62.5, and 25 μg/mL). One‐way ANOVA for (A, B, E, F) and two‐way ANOVA for (C) & Dunnett's multiple comparison test with the untreated group. CM: 62.5 μg/mL chloramphenicol.

We have shown that SWe affects key regulators and reduces *in vitro* biofilm and virulence phenotypes of *S. aureus* SH1000, but the regulations of virulence phenotypes (e.g., intactness of Agr system) and biofilm formation (e.g., PIA dependency) are diverse among strains. We tested the effect of SWe on two other strains, MW2 and ATCC25923. ATCC25923 is known for the medium biofilm formation and the lack of Agr function (Tan et al. 2015), in contrast, MW2 is a weak biofilm former with intact Agr function (Morales‐Laverde et al. 2022; Trotonda et al. 2008). Our assay confirmed these biofilm phenotypes (Figure 5A). SWe significantly reduced the biofilm of ATCC25923 but not of MW2 (Figure 5A). The anti‐biofilm effect against ATCC25923 was not due to the growth inhibition (Figure 5B). Thus, SWe can disturb biofilm irrespective of the status of the Agr function.

In MW2, SWe suppressed the transcription of the RNA‐III, as shown by the reporter assay (Figure 5C). Moreover, we also confirmed that SWe reduces hemolytic activity, colony spreading, and pigmentation (Figure 5D–F). These results suggest that SWe can reduce the *in vitro* virulence phenotypes of MW2 without enhancing the biofilm formation. Overall, SWe can disturb the Agr system and impair biofilm in any type of strain tested. The effect of SWe on different strains is summarized in Table 3.

**Table 3:** Summary of the effect of SWe on *S. aureus* strains.

Strain	Characteristics	Viability	Biofilm	Agr‐dependent virulence			Pigmentation
				RNA‐III reporter	Hemolysis	Colony spreading	
ATCC25923	MSSA, *agr* type III (inactive), moderate biofilm	→	↓	not tested	not tested	not tested	not tested
SH1000	MSSA, *agr* type I, moderate biofilm	→	↓	↓	↓	↓	↓
MW2	MRSA, *agr* type III, weak biofilm	→	→originally weak	↓	↓	↓	↓

## 3 Discussion

SWe may not be immediately suitable for direct clinical use, as is the case with many natural plant products, and the present *in vitro* study under limited conditions does not necessarily translate to what occurs in infection. The significance of this study lies in providing the first example of a potential solution to overcome the anticipated limitations of current anti‐quorum‐sensing strategies against *S. aureus*. Inhibition of Agr can be achieved by a variety of inhibitors with different modes of action (Vinodhini and Kavitha 2024). These anti‐Agr activities are generally not through bactericidal activities or growth inhibition, which is an important feature for anti‐virulence drugs that would not generate drug resistance. However, the inhibition of Agr sometimes results in increased biofilm. For example, staquorsin, which is designed to interfere with the AgrA, promotes the biofilm formation of Newman (Mahdally et al. 2021). The extract of *Schinus terebinthifolia* fruit (450D‐F5), and the purified compound (termed compound 1, oleanolic acid family) inhibit Agr and have a dose‐dependent effect on biofilm but increase biofilm at low concentrations (Muhs et al. 2017) (Tang et al. 2020). Thus, no report has clearly shown the inhibition of both biofilm and Agr without affecting the growth. In this context, the present study first reported an example where the cells with suppressed Agr activity can sustain their planktonic lifestyle rather than shifting into the biofilm status, with the information about changes in the biofilm matrix and transcriptome underlying this interesting cellular status.

Regarding biofilm matrix compositions, SWe reduced eDNA (Figure 2B). The eDNA is the universal component in biofilm matrix not only in bacteria but also in fungi, with a negative charge in the structure to connect tightly to other molecular components in ECM (Campoccia et al. 2021; Liu et al. 2022; Shopova et al. 2013). Targeting eDNA for biofilm treatment has been focused on due to such a critical role (Campoccia et al. 2021). Previous studies have shown the relationship between eDNA and autolysis during biofilm formation (Biswas et al. 2006; Mann et al. 2009; Rice et al. 2007). We also found that SWe reduces autolytic activity (Figure S6), though the transcriptome analysis did not directly explain this. The SaeRS is known to affect the expression of *atl* encoding major autolysin (Mashruwala et al. 2017) and *nuc* encoding staphylococcal thermonuclease (Liu et al. 2016; Olson et al. 2013). However, the *atl* and *nuc* expressions were not changed by SWe. It might be valuable to note that autolysin can be inactivated by the local acidification at cell wall areas that are caused by the augmented respiration (Biswas et al. 2012). Cell wall metabolism and its regulator would also affect the cell lysis; for example, an alternative sigma factor SigB affects the cell wall thickness and changes the sensitivity to lysostaphin (cell wall degrading enzyme) (Morikawa et al. 2001). The present finding that SWe reduces the expression of *asp23* that is representative of the SigB regulon (Figure S17) may have implications in this aspect.

In addition to the decrease in eDNA, SWe increased PIA (Figure 2A), and altered the expression of genes encoding adhesin/cell surface proteins (Figure 4A). These qualitative changes in the biofilm matrix are likely the underlying mechanism in the SWe‐dependent reduction in biofilm. Several virulence factors like Hla, Hld, and PSMs also contribute to the biofilm structure of *S. aureus* (Graf et al. 2019; Schwartz et al. 2012; Tayeb‐Fligelman et al. 2017). SWe affected the expression of these factors in Agr‐positive strains, SH1000 and MW2 (Figure 5).

The present study found that SWe negatively affects the SaeRS system. The SaeRS system has been known as the critical regulator of biofilm (Mashruwala et al. 2017; Moormeier and Bayles 2017; Mrak et al. 2012) (Figure S1). Some reports showed that SaeRS targeting compounds reduce biofilm in *S. aureus*. An example that inhibits SaeR is Fenoprofen, a nonsteroidal anti‐inflammatory drug (trade name Nalfon) (Jiang et al. 2023). Fenoprofen can restore the walking of mice with implant‐associated osteomyelitis. The Fenoprofen‐treated biofilm structure was loosened and porous due to the reduction of eDNA and proteins. The PIA production was unchanged, and its antibiofilm activity was not recorded in the PIA‐dependent biofilm type, unlikely in our SWe case.

One interesting question raised by this study is what happens to the cellular status when both Agr and Sae are inhibited simultaneously. While the expression of many toxin‐related genes would likely be impaired, what would happen to the biofilm? We plan to investigate this in our future study.

The carotenoid pigments are among the virulence factors to cope with oxidative stress (Clauditz et al. 2006). The expression of genes encoding CrtMN carotenoid synthesis enzymes is controlled by the alternative sigma factor SigB (Xue et al. 2019). Our RNA‐seq analysis detected the SWe‐dependent reduction in expression of *asp23* (representative of SigB regulon) at 3 h (Table S2, Figure S17), but not in *crtMN*. Several studies have shown the involvement of some signaling and metabolism in pigmentation, e.g., Agr system, respiration, and metabolic pathways (Lan et al. 2010) (Schurig‐Briccio et al. 2020) (Marroquin et al. 2019). SWe‐dependent reduction in pigmentation is likely a part of the alteration in cellular metabolism and signaling, where ROS and the Agr system are involved. Alternatively, some molecules in SWe might be inhibitors of carotenoid synthesis enzymes, likely as the suggested mechanism of farnesol inhibition of carotenoid synthesis (Vila et al. 2019).

The methanol and ethanol extract of sappanwood had bactericidal activity but no anti‐QS activity (data not shown), unlikely to the DCM solvent used in this study (SWe). This suggests that the anti‐QS compound(s) in the DCM extract has less polarity. Sappanwood contains a variety of compounds, including xanthone, coumarin, phenolic compounds, chalcones, flavones, homo isoflavonoids, and brazilin (Vij et al. 2023). Peng et al. show the antibiofilm effect of brazilin by upregulating *icaR* and *agrA* expression (Peng et al. 2018), which is different from the effect of SWe in this study. Sappanwood extracted in DCM may also contain fatty acids (Ngernnak et al. 2018), which can show antibiofilm activity (Alenazy 2023; Lee et al. 2017). Bioactive compound(s) in SWe are to be identified in our ongoing studies to understand the molecular input(s) that suppress *in vitro* virulence phenotypes and biofilm. In addition, exploring the other extracts or compounds that can suppress both Agr and biofilm, followed by the analysis of the cellular status, would be an important comparative study to unravel the general cellular response that can maintain *S. aureus* in this status.

## 4 Materials and Methods

### 4.1 Extraction From *B. sappan*

*B. sappan* wood was purchased from a Traditional medicine pharmacy in Ho Chi Minh City. It was finely ground into a powder. Ten grams of the powder were soaked in 100 mL of Dichloromethane (DCM) (270997, Merck, Germany) solvent and allowed to extract for 24 h. The extract was then filtered by Whatman filter paper to collect the liquid phase. This DCM extraction process was repeated twice, combining the resulting extracts. The solvent was subsequently evaporated to yield the DCM extract. The obtained extract was dissolved in 100% dimethyl sulfoxide (DMSO) (4987481246928, FUJIFIRM Wako, Japan) to achieve 50 mg/mL, which served as the stock solution for subsequent experiments (designated as SWe). We used this concentration as the approximate reference; SWe still contains DMSO‐insoluble materials, including the wood powder. Insoluble materials were removed by 0.22 µm membrane filter (for SWe lot number 1) (Minisart‐NY25 17845 ACK, Sartorius, Germany), or centrifugation at 10,000 rpm for 3 min (for SWe lot number 2). The extract was checked for its antibiofilm activity. The ones with confirmed antibiofilm and anti‐Agr activities were selected and used in this study (lot numbers 1 and 2). SWe‐lot1 is used for all main experiments, and SWe‐lot2 (SWe^l^°^t2^) was used in the Supporting Figures S3, S5, S7, S10, S12, S15, and S16 since SWe‐lot1 was not available anymore.

### 4.2 Bacterial Strains and Culture Conditions

The strains used in this study are listed in Table 4. Strains SH1000, MW2 and ATCC25923 are from the laboratory stock collection. *S. aureus* SH1000 served as the model strain to explore the anti‐biofilm and anti‐virulence properties of SWe. The deletion mutant of the *agr* locus (SH1000Δagr) was constructed by using pMAD‐tet‐Δagr (Gor et al. 2019). Cells were inoculated in Tryptic Soy Broth (TSB) (00382902571612, BD, USA) or Mueller Hinton Broth (MHB) (00382902757306, BD, USA) from −80°C glycerol stocks and grown at 37°C with shaking at 180 rpm overnight. Chloramphenicol (12.5 μg/mL) (036‐10571, FUJIFILM Wako, Japan) was supplemented to TSB for the overnight cultures of reporter strains, SH1000‐RNA III‐gfp and MW2‐RNA III‐gfp.

**Table 4:** List of *S. aureus* strains and overnight culture condition.

Strain	Characteristics	Overnight culture in	Reference
ATCC25923	MSSA, *agr* type III (inactive), moderate biofilm	TSB	Tan et al. (2015)
SH1000	MSSA, *agr* type I, moderate biofilm, drived from 8325	TSB	Horsburgh et al. (2002)
MW2	MRSA, *agr* type III, weak biofilm	TSB	CDC (1999)
SH1000 P3*venus*	SH1000 carrying the pRIT_RNAIII_ *venus* Agr reporter plasmid	TSB + 12.5 μg/mL chloramphenicol	This study
MW2 P3*venus*	MW2 carrying the pRIT_RNAIII_ *venus* Agr reporter plasmid	TSB + 12.5 μg/mL chloramphenicol	This study
SH1000Δagr	SH1000 deletion mutanto of *agr* locus.	TSB	This study
SA113	MSSA, *rsbU‐*, drived from 8325	TSB	Iordanescu and Surdeanu (1976); Bæk et al. (2013)

### 4.3 MIC

The MIC value was determined following the protocol of the CLSI standard (“CLSI M26‐A ‐ Methods for Determining Bactericidal Activity of Antimicrobial Agents; Approved Guideline, ”n.d.). Bacteria cultured overnight in MHB were diluted to a 10^6^ CFU/mL concentration. A volume of 100 μL of the bacterial suspension (10^5^ CFU) was distributed into each well of a 96‐well microtiter plate. Successive two‐fold dilutions of the extract SWe, ranging from 2000 μg/mL (4% volume of SWe) to 0 μg/mL, were prepared in MHB. Subsequently, 100 μL of each diluted extract was added to the corresponding wells. DMSO, at a concentration of 4%, served as the solvent control and was diluted similarly to the extract. Bacterial cultures without any additives were also tested. The microtiter plate was incubated at 37°C for 24 h. The MIC value was determined as the lowest concentration of the extract that inhibited visible bacterial growth.

### 4.4 Biofilm Assay and Viability Test (96‐well Plates)

The biofilm assay protocol was adapted from Gomes et al. (Gomes et al. 2019). *S. aureus* was cultivated in 96‐well plates (260860, Thermo, Denmark) (Figures 1, 2, 5). Each well contained 100 μL of TSB and 10^6^ CFU of *S. aureus*. Appropriate concentrations of SWe were supplemented. Control groups included TSB alone, TSB with DMSO solvent controls (1% DMSO is equivalent to SWe at 500 µg/mL prepared from 50 mg/mL stock), and TSB with 62.5 µg/mL of Chloramphenicol. Each condition was tested in triplicate in each independent experiment. The plates were incubated at 37°C for 24 h. After incubation, planktonic bacteria were removed, and the wells were washed twice with phosphate‐buffered saline (PBS). Biofilms were fixed by adding 100 μL of 90% ethanol to each well. After 2 min incubation, the ethanol was removed and the plate was air‐dried. The biofilms were stained with 0.1% crystal violet solution for 5 min, after which the stain was removed, and the wells were air‐dried and washed with PBS. The absorbed crystal violet dye in biofilms was solubilized in 100 μL of 90% ethanol for 15 min on the horizontal shaker. The harvested crystal violet was measured as absorbance at 570 nm.

To evaluate the effect of SWe on SH1000 growth (Figure 1C), both the supernatant and biofilm were collected after 24 h of incubation. The biofilm structures were disrupted by pipetting. CFU was counted by using TSB agar (TSA) plates.

### 4.5 Biofilm Assay and Viability Test (Optimized for 24‐Well Plates)

*S. aureus* was cultivated in 24‐well plates (3526, Corning, USA) (Supporting Figures S5, S11). Each well contained 300 μL of TSB and 3 × 10^6^ CFU of *S. aureus*. Planktonic bacteria were removed, and wells were washed twice with phosphate‐buffered saline (PBS). To evaluate the effect of SWe on cell growth (Figure S11), both the supernatant and biofilm were collected after 24 h of incubation.

For CV staining (Figure S5), protocol was optimized for the 24‐well plate system. Washed biofilms were first air‐dried and fixed by adding 500 μL of 80% ethanol to each well. After 2 min incubation, ethanol was diluted in a stepwise manner (80%, 40%, and 5%). The biofilms were stained with 0.1% solution of crystal violet (031‐07331, FUJIFILM Wako, Japan) for 5 min. The stained biofilm was washed once with water, and the plate image was scanned. The absorbed crystal violet dye in biofilms was solubilized in 600 μL of 90% ethanol with 1.5% HCl. The harvested crystal violet was measured as absorbance.

### 4.6 Polystyle Binding Assay

Overnight culture of SH1000 in TSB was diluted 200‐fold in fresh TSB. 450 µg/mL SWe^l^°^t2^ or DMSO solvent control was supplemented, and each sample was distributed to 4 wells (quadruplicate) of the 96‐well plate (260860, Thermo, Denmark) as in the biofilm assay. The plate was kept at 37°C for 30 min and then washed three times with PBS. Fresh 100 µL/well TSB was added to the washed plate, and the increase in OD_595_ was measured by Infinite F Nano+ plate reader (Tecan, Switzerland) at 37°C with shaking. The time to reach OD_595_ = 0.2 was used to estimate the CFU using standard samples (overnight culture of SH1000) with known CFU values.

### 4.7 Quantification of ECM Components

The extraction and quantification of the ECM were conducted following a modified version of the method described by Chiba et al. (Chiba et al. 2015). Biofilms were collected from 96‐well plates and dissolved in 1.5 M NaCl, followed by a 5‐min incubation at room temperature. The samples were then centrifuged at 5000 rpm for 10 min to separate the supernatants. These supernatants were subsequently analyzed for PIA content, eDNA, and protein concentration.

PIA was quantified using Wheat Germ Agglutinin (WGA), a carbohydrate‐binding lectin with a high affinity for sialic acid and N‐acetylglucosamine. This lectin stains yeast buds as well as the cell surfaces of gram‐positive bacteria and animal cells (Wang et al. 2016). A 5 μL aliquot of each sample was dot‐blotted onto a nitrocellulose membrane. The membrane was blocked with 1% Bovine Serum Albumin (BSA) and incubated with HRP‐WGA reagent (29073, Biotium, USA) at a concentration of 130 ng/mL in 1% BSA solution. Detection of PIA was performed using an Enhanced Chemiluminescence (ECL) solution (1705060, Bio‐Rad, USA) and visualized with a FUSION chemiluminescence, fluorescence, and gel imaging system (Vilber Lourmat, Germany). The signal of EPS was processed by ImageJ software, then the EPS (%) was calculated: (signal/area)_treated_/(signal/area)_untreated_ x 100.

eDNA was quantified using the Quant‐iT PicoGreen dsDNA Reagent and Kit (P11496, Thermo Fisher, USA) according to the manufacturer's instructions. Protein concentration was measured using the DC Protein Assay Kit I (5000111, Bio‐Rad, USA).

### 4.8 PNAG Detection by Using DspB and Its Derivatives

PNAG was detected by DspB‐based PNAG binding assay (filter method) and by microscopic observation as described by Eddenden & Nitz (Eddenden and Nitz 2022) with some modifications. DspB is the enzyme that degrades PNAG. DspB^E184Q^ binds to PIA but does not degrade PNAG. DspB^E184Q W330Y^ does not bind PIA. The GFP fusion proteins with these DspB variants were used as the PNAG probe and the negative control, respectively.

Plasmids were obtained from Addgene and transformed into *E. coli* BL21(DE3). The plasmid pET29b‐dspBwt‐6xHis was generated from pET29b‐dspB(E184Q)‐6xHis by inverse PCR and transformed into BL21(DE3). Recombinant proteins were purified as described (Eddenden and Nitz 2022) by using TALON Metal Affinity Resin (Takara). The harvested proteins were concentrated using Amicon Ultra‐0.5 centrifugal filters (3 kDa MWCO, Millipore), and the buffer was exchanged to PBS. Protein concentrations were determined using DC protein assay kit (Bio‐Rad).

SH1000 cells were statically grown at 37°C in TSB with or without 450 µg/mL SWe^lot2^ or DMSO solvent control. Cells were harvested after 24 h, washed with 0.1% BSA in PBS, and submitted to the following filter method or microscopic observation.

For the filter method, cell density was adjusted to an OD_600_ of 1.5. A 150‐μL cell suspension was applied to a Corning Costar Spin‐X centrifugal filter tube (0.45 µm, cellulose Acetate) and centrifuged at 6000 × g for 10 min at 4°C. Retained cells were suspended with 150 μL of 0.1% BSA in PBS and incubated at room temperature for 5 min. After centrifugation, cells were suspended in 150 μL of 0.05 μM PNAG‐binding probe and incubated at room temperature for 30 min. At each of the following steps, solutions passed through the filter by centrifugation were kept for the GFP fluorescence measurement. The cells on the filter were washed with 150 μL of 0.1% BSA in PBS and reacted with the 150 μL solution of 0.25 μM DspBwt and 0.1% BSA in PBS at room temperature for 1 h, followed by the final centrifugation. The fluorescence intensity of each collected solution was measured using excitation and emission wavelengths of 470 and 515 nm.

For microscopic observation, washed cells derived from 50 µL overnight culture were suspended in 200 μL of 0.05 μM PNAG‐binding probe and kept for 10 min at room temperature. The DNA was stained with Hoechst 33342 (Invitrogen) for 15 min at room temperature. After washing with PBS twice, samples were observed using a fluorescence microscope (Olympus BX53). Images were analyzed using ImageJ software (1.54p, (Schindelin et al. 2012)).

### 4.9 Bacterial Lysis Assay

The overnight culture of SH1000 was diluted 100‐fold in TSB. SWe (final concentration of 250 μg/mL) was added to 5 mL of the prepared bacterial suspension in a 15 mL tube. Tubes were incubated at 37°C with shaking at 180 rpm for 3 h, after which the bacterial cells were harvested by centrifugation at 10,000 rpm for 2 min at room temperature. The resulting pellets were washed twice with PBS. Subsequently, the pellets were suspended in 1 mL PBS and adjusted to OD_600_ of 0.2–0.3. A volume of 100 μL of each adjusted suspension was added to a 96‐well plate as triplicate for each sample. The OD_578_ was monitored every 15 min for a total of 3 h with shaking at 180 rpm.

### 4.10 DNase I, Protease K, and Metaperiodate Treatment of Biofilm

Biofilm stability against DNase I (LS002138, Worthington, USA), Protease K(166‐28913, FUJIFILM Wako, Japan), and sodium metaperiodate (1.06597, Merk, Germany) was tested as previously described (Seidl et al. 2008) (Sugimoto et al. 2018) with some modifications. The cells were inoculated in a 24‐well plate (300 µL TSB medium per well) and incubated at 37°C. After 24 h, DNase I (140 U/mL, final conc.), Protease K (100 µg/mL), or sodium metaperiodate (10 or 50 mM) (Seidl et al. 2008) was directly added to each well, followed by an additional 2 h of incubation at 37°C.

In separate wells, DNase I was added to the culture upon inoculation, and the biofilm formation in the presence of DNase I was observed (Figure S5, right column).

Following the treatment, biofilm was quantified by CV as described above (optimized method for 24‐well plates).

### 4.11 Effect of Extract Treatment on Hemolytic Activity

An overnight culture of *S. aureus* SH1000 was diluted to approximately 10^7^ CFU/mL in TSB supplemented with or without SWe and the solvent control 1% DMSO. Cultures were incubated at 37°C with shaking at 180 rpm for 16–18 h. After incubation, cells were pelleted by centrifugation at 10,000 rpm for 3 min, and the supernatants were collected. A 250 µL aliquot of each supernatant was mixed with 250 µL of a 2% sheep red blood cell suspension prepared in PBS 1X. The mixtures were incubated for 1 h at 4°C, followed by centrifugation at 500 rpm for 5 min to pellet intact red blood cells. Hemolytic activity was quantified by measuring the absorbance of the supernatants at 540 nm.

### 4.12 Effect of SWe on Colony Spreading

Colony spreading was performed as described by Kaito et al. (Kaito and Sekimizu 2007). SH1000 was cultured at 37°C in TSB for 16–18 h with shaking at 180 rpm. Subsequently, 2 μL of the bacterial culture was spotted onto 0.24%‐agar TSA plates supplemented with SWe at MIC, 1/2 MIC, and 1/5 MIC. Control groups included no SWe or 1% DMSO (solvent control). All plates were incubated at 37°C for 16–18 h.

### 4.13 Carotenoids Measurement

The carotenoid pigments were extracted and quantified as described (Morikawa et al. 2001). An overnight culture of SH1000 was diluted to 10^7^ CFU/mL in TSB supplemented with SWe. Negative controls consisted of untreated bacteria and those treated with 1% DMSO. Cultures were incubated at 37°C for 16–18 h with shaking at 180 rpm. Following incubation, cells were harvested by centrifugation at 10,000 rpm for 1 min, and washed twice with sterile water at room temperature. The cells were suspended in 500 μL of methanol and incubated at 55°C for 5 min. After centrifugation, the supernatants were collected. The methanol extraction was repeated once more. The absorbance of the combined supernatants was measured at 465 nm using a plate reader with a 100 μL sample. The percentage of pigmentation was calculated relative to the untreated control group.

### 4.14 Fluorescence Reporter Assay of Agr Activity

Overnight cultures of SH1000 P3*venus* and MW2 P3*venus* reporter strains were diluted to 10^7^ CFU/mL in TSB supplemented with SWe. Controls are TSB or TSB supplemented with 1% DMSO. A 100 μL aliquot of each bacterial suspension was added to 96‐well plates in triplicate. The OD at 590 nm and fluorescence (FL) signal (485–520 nm) were measured using an Infinite F Nano+ plate reader (Tecan, Switzerland) every 15 min for 25 h with an orbital shaking amplitude set of 4.

### 4.15 RNA Extraction for qPCR and RNA‐Seq

Cells were grown under static conditions as previously described. At 3, 9, and 24 h, the cell suspensions, including both planktonic and biofilm cells, were harvested in new tubes and centrifuged at 10,000 rpm for 3 min at 4°C. The cell pellets were washed twice with suspension buffer (50 mM Tris, pH 8.0, 1 mM EDTA, 50 mM NaCl) and resuspended in 200 μL of the same buffer. Five microliters of 10 mg/mL lysostaphin (120‐06611, FUJIFIRM Wako, Japan) was added to the suspension and incubated at 37°C for 10 min, followed by the addition of 1 mL ice‐cold TRIzol reagent (93289‐100 ML, Sigma, USA). The mixture was incubated on ice for 5 min and was centrifuged at 13,000 rpm for 20 min at 4°C. The upper aqueous phase was collected, and 200 μL of cold chloroform was added. The mixture was vortexed at maximum speed for 3 min, left for 3 min to facilitate phase separation, and centrifuged at 13,000 rpm for 15 min at 4°C. The upper phase was collected and mixed with an equal volume of cold isopropanol. The mixture was incubated at ‐20°C for 20 min, followed by centrifugation at 15,000 rpm for 20 min at 4°C to pellet the RNA. The RNA pellet was washed twice with cold 70% ethanol, air‐dried for 10 min, and dissolved in 30 μL of DEPC‐treated water. The extracted RNA was quantified by NanoDrop™ OneC UV‐Vis Spectrophotometer (Thermo Fisher, USA).

### 4.16 qPCR

Following RNA extraction, the samples were treated with DNase and reverse transcribed into cDNA using the PrimeScript RT reagent Kit with gDNA Eraser (RR047A, Takara, Japan) according to the manufacturer's instructions. TB Green Premix Ex Taq II Fast qPCR kit (RR830A, Takara, Japan) was used for qPCR. Three separate samples were each tested in triplicate. The relative fold change in gene expression was determined using the 2^–∆∆Ct^ method using 16S RNA as the normalization reference. The primers used for detecting gene expression are listed in Table 5.

**Table 5:** Primer list for qPCR.

No.	Primer	Sequences	Product size (bp)
1	agrA_F	GT CTA CAA AGT TGC AGC GA	101
2	agrA_R	GTA AGC GTG TAT GTG CAG T	
3	hld_F	TAA GGA AGG AGT GAT TTC AAT GG	91
4	hld_R	AGT GAA TTT GTT CAC TGT GTC GAT	
5	hla_F	TGG AAC CCG GTA TAT GGC AA	122
6	hla_R	GCG AAG TCT GGT GAA AAC CC	
7	psma_F	TGG GTA TCA TCG CTG GCA	102
8	psma_R	ACC CAT GTG AAA GAC CTC CT	
9	psmb_F	CGT TGA AAA TGG CGT AGG TT	166
10	psmb_R	TCA CAC TAT CAT GTT GTT GTG C	
11	saeR_F	TCG TGG ATG ATG AAC AAG AC	104
12	saeR_R	TTA CCG CTA GTT GTC GTT GT	
13	saeP_F	TCG CAA TGG TTG ACT ACG AT	112
14	saeP_R	CCG AAG ATG ACG TGA GCT TT	
15	16S_F	CGT AGG TGG CAA GCG TTA TC	108
16	16S_R	GTT TCC AAT GAC CCT CCA CG	
17	luxS_F	GCTACTGAAGTGCCTGCTTG	105
18	luxS_R	TGT ACC GAA AAC ATC ATG CCA	
19	kdpDE_F	TAT AAA CCG AAC CAA CCA CA	116
20	kdpDE_R	GAT TGC GTG ATC ATC TTC AA	
21	capAB_F	AGG ATG GTT GCT ACT TAT GTC	110
22	capAB_R	TGT TTG AAC GTA TAC CTC GA	

### 4.17 RNA‐Seq

RNA‐seq was done by Rhelixa company (Japan). rRNA was removed from total RNA using a RiboZero Plus rRNA Depletion kit (Epicentre, USA). Transcripts were fragmented and used as a template to generate a strand‐specific cDNA library using the NEBNext Ultra II Directional RNA Library Prep kit (NEB, USA). The sequencing was conducted on the Illumina NovaSeq. 6000 system with 150 bp paired‐end reads. RNA‐seq raw reads were trimmed by Trimmomatic software and aligned to chromosomes of *S. aureus* SH1000 (NZ_CP059180.1) by HISAT2 (Kim et al. 2018). Mapped reads were counted using ‘featureCounts’ (Liao et al. 2014). Differentially expressed genes between the SWe‐treated sample and untreated sample were analyzed with edgeR (Chen et al. 2014) with the threshold *p* < 0.05 (Benjamini‐Hochberg method) and |log_2_foldchange | > 1.

### 4.18 Fibronectin Binding Assay

Fibronectin binding assay was carried out as described in (Gries et al. 2020) with some modifications. High‐protein‐binding 96‐well plates (Costar) were coated with 10 μg/mL human fibronectin (Merck) or 1% BSA at 4°C overnight. The solutions were removed, followed by blocking with 2% BSA for 1 h at room temperature and washing with PBS three times.

SH1000 cells were grown in TSB with or without 450 µg/mL SWe^lot2^ or DMSO solvent control for 4 h in 24‐well plates. Harvested cells were washed with PBS and suspended in PBS to a density of OD_595_ = 1.0. 100 µL of the cell suspension was added to the well and incubated at 37°C for 1 h. Non‐adherent cells were removed, and the plate was washed with PBS three times. The adhered cells were fixed with 4% paraformaldehyde in PBS for 15 min and washed twice with PBS. Fixed adherent cells were stained with 0.5% crystal violet in 12% EtOH for 3 min, washed four times with PBS, and the bound dye was eluted with 0.1 mL 7% acetate. The eluted dye was quantified as OD_595_ and normalized against bacterial binding to the wells coated only with BSA.

### 4.19 NO and ROS Detection

The biofilm cells at 9 and 24 h were harvested and centrifuged at 15,000 rpm for 2 min at room temperature. The cells were washed once and resuspended in 1 mL of PBS. For plate reader analysis, the cell pellet was adjusted to OD_600_ of approximately 0.2 and divided into two tubes, each containing 500 μL, to separately detect ROS and NO. To detect intracellular ROS, CellROX Orange Reagent (C10443, Thermo Fisher, USA) was added to a final concentration of 5 μM and incubated for 30 min at 37°C in the dark. For NO detection, 5 μM DAF‐FM diacetate reagent (16296, Cosmo Bio, Japan) was added and incubated for 1 h under the same condition. After incubation, cells were pelleted and washed twice with PBS. The cells were resuspended in 500 μL of PBS, and ROS and NO levels were measured at 545–565 nm and 485–520 nm, respectively, using Infinite® M Nano (Tecan) plate reader.

For microscopy analysis, DAF‐FM diacetate reagent was added to the cell suspension at a final concentration of 5 μM and incubated for 30 min at 37°C in the dark. Subsequently, CellROX Orange Reagent was added to the same tube at the final concentration of 5 μM and further incubated for 30 min. Cells were washed and resuspended in 1 mL of PBS. Two microliters of the suspension were loaded onto a glass slide and observed under a fluorescent microscope (BZ‐X710, Keyence).

### 4.20 Statistical Analysis

All assays were repeated by at least three independent experiments. Statistical analysis was performed with GraphPad Prism (version 8.0) software (La Jolla, CA). Statistical methods are shown in figure captions.

## Author Contributions

**Nghi Bao Nguyen:** conceptualization, formal analysis, visualization, writing – original draft, writing – review and editing, investigation, methodology, validation. **Yuri Ushijima:** supervision, resources, writing – review and editing, investigation. **Raiku Sekiya:** writing – review and editing, investigation, methodology. **Kazuya Morikawa:** conceptualization, funding acquisition, project administration, resources, supervision, visualization, writing – original draft, writing – review and editing, investigation, methodology, validation. **Le Thuy Thi Nguyen:** project administration, resources, supervision, visualization, writing – review and editing, investigation, methodology.

## Ethics Statement

The authors have nothing to report.

## Conflicts of Interest

The authors declare no conflicts of interest.

## Supporting information


**Figure S1:** Biofilm development and factors involved. Biofilm development and the factors involved are well summarized in review articles (Moormeier and Bayles 2017; Paharik and Horswill 2016). In the attachment and multiplication stages, the microbial surface components are involved in cell adherence and cell‐to‐cell interactions. The involved factors are EPS (PIA) and cell wall‐associated proteins such as staphylococcal protein A (SpA), fibronectin‐binding proteins (FnBPA, FnBPB), collagen‐binding protein, and clumping factors (ClpA, ClpB). The eDNA released by cell death contributes to biofilm stabilization by serving as a “glue” between cells via interaction with extracellular and cell wall‐associated proteins and PIA in the biofilm matrix (Campoccia et al. 2021). The exodus stage precedes the maturation of biofilm. The biofilm is reconstructed through the early dispersal by the nuclease (Nuc) expression in the subpopulation that destroys the eDNA. The Nuc expression is under the control of the SaePQRS system (Liu et al. 2016). The exodus stage may contribute to architecting the mature biofilm to enhance nutrient exchange and waste removal (Moormeier et al. 2014). In the dispersal stage, exoenzymes and phenol soluble modulins (PSMs) disseminate cells to distal sites (Boles and Horswill 2008) (Periasamy et al. 2012). PSMs also forms amyloid or complex with DNA, which constitute the biofilm (Howard et al. 2023) (Grando et al. 2022) (Schwartz et al. 2012), or compact and thicken biofilm.
**Figure S2:** Key virulence regulators: Agr system and SaeRS system. The Agr system is encoded by two regions under the control of P2 and P3 promoters. The *agrBDCA* operon encodes the quorum sensing system. The *agrD* gene encodes the peptide precursor for AIP. AgrD is then modified and exported by the transmembrane endopeptidase AgrB. The *agrC* and *agrA* genes encode the two‐component system to sense the AIP concentration that increases in high cell density or closed environment such as in phagosomes (thereby also known as diffusion sensing system). The histidine kinase sensor AgrC activates AgrA by phosphorylation. The activated AgrA directly upregulates the P2 and P3 promoters and expression of PSMs. The P3 promoter controls the transcription of the RNA‐III factor. RNA‐III itself encodes δ‐hemolysin (*hld*). By forming RNA duplexes, RNA‐III also acts as a translational regulator. It directly or indirectly (*via* Rot) activates the expression of exoenzymes such as nuclease, lipases, and leukocidins, and blocks that of a series of surface proteins (SpA, FnBPs, etc). The *S. aureus* exoprotein expression (Sae) SaeRS system is the two‐component system regulating the expression of virulence factors including toxins and immune‐evasive proteins (Liu et al. 2016). SaeRS regulon includes α‐, β‐ and γ‐hemolysins, coagulase, Panton‐Valentine leucocidin (LukGH), TSST‐1, enterotoxin‐like toxin, nuclease and adhesins (Eap, FnbAB, Efb, Eab, etc.). The *saePQRS* operon is under the control of two promoters, P1 and P3. Human neutrophil peptides (HNP 1 to 3), calprotectin, hydrogen peroxide, and subinhibitory concentrations of β‐lactam antibiotics are reported to activate the SaeRS. On the other hand, several factors, such as low pH conditions, high concentrations of NaCl, or some fatty acids, affect it negatively. The activated SaeR leads to the autoactivation of the promoter P1. SaePQ also controls this autoactivation. Moreover, Agr system and Rot are involved indirectly in the regulation of *saePQRS*.
**Figure S3:** SWe^lot2^ does not affect initial attachment to the 96‐well plate. Overnight culture of SH1000 was diluted 200‐fold in TSB. 450 µg/mL SWe^lot2^ or DMSO solvent control were supplemented and distributed to the 96‐well plate (quadruplicate). The plate was kept at 37°C for 30 min. The plate was washed three times by PBS. Fresh TSB was added to the washed plate, and the increase of OD595 was measured by plate reader. The time to reach at OD595 = 0.2 was determined, and the CFU was estimated by calculation using a standard sample (overnight culture of SH1000) whose CFU value was counted by the plating method.
**Figure S4:** The antibiofilm activity of SWe on SH1000 in BHI + NaCl3% (EPS‐inducing media) after 24 hours of treatment. (A) Cell viability in total biomass (biofilm and planktonic cells) relative to the untreated sample. The viable cells were counted as CFU. (B) Biofilm was quantified using crystal violet, and the values of percent change relative to the untreated group are shown. Error bars indicate the SD. *p ≤ 0.05, **p ≤ 0.001, ***p ≤ 0.0001 compared to the untreated group. One‐way ANOVA & Dunnett's multiple comparison test with the untreated group. CM: 62.5 μg/mL chloramphenicol. The 250 µg/mL SWe reduced biofilm without significant growth inhibition, but 500 µg/mL SWe reduced the viability and its antibiofilm activity is not clear.
**Figure S5:** SWe^lot2^ increases PIA production. PIA(PNAG) was quantified by filter method **(A)** and by microscopic observation **(B, C)** using DspB variants. DspB is the enzyme that degrade PNAG. DspB^E184Q^ binds to PIA, but does not degrade PNAG. DspB^E184Q W330Y^ does not bind PIA. The GFP fusion of these DspB variants were used as the PNAG probe, and the negative control, respectively. Cells were harvested after 24 h static growth, and submitted to each assay as described in Methods. 450 µg/mL SWe^lot2^ was used in these experiments. **(A)** Cells pretreated with PNAG probe or control were washed and treated with DspB enzyme to degrade PNAG. The release of the probe by PNAG degradation was quantified. Mean and SD are shown (n = 2). *p = 0.0133, Dunnett's multiple comparison test. **(B)** Cells were mixed with DspB^E184Q^‐GFP or DspB^E184Q W330Y^ –GFP and Hoechst 33342. Mean and SD of the percentages of GFP positive cells from three independent experiments are shown. ***p = 0.0008, *p = 0.0318, Dunnett's multiple comparison test. **(C)** Representative images. These data indicates that SWe increases PIA, but it may also increase certain substances that can bind to DspB^E184Q W330Y^ ‐GFP (B).
**Figure S6:** Autolysis of SH1000 reduced by SWe. Cells were pretreated with SWe or 1% DMSO for 3 h, and washed before the assay. The autolysis assay was performed in the shaking condition at 37^o^C in a 96‐well plate. OD_578_ was measured by the plate reader.
**Figure S7:** Biofilm disruption assay. Cells were statically cultured with or without SWe (lot number 2) in a 24‐well plate. DMSO is the solvent control (0.9%, same with 450 µg/ml SWe). After 24 h incubation, DNase I, Protease K, or metaperiodate were added directly, without refreshing the medium. In the right column, DNase was added at the beginning and the biofilm was formed in the presence of DNase. Biofilm was quantified by the optimized crystal violet method and normalized to the non‐treated biofilm (left top). Mean (bold) and ±SD values are shown (n = 2).
**Figure S8:** Microscopy images of SH1000 P3*venus* reporter strain after 20 h incubation with SWe. Fluorescent cells were counted, and the average percentage of fluorescent cells was shown. The error bars show SD (n = 2).
**Figure S9:** Downregulated and upregulated genes shared among 3 h, 9 h, and 24 h. Red, green, and blue indicate 3 h, 9 h, and 24 h after exposure to SWe.
**Figure S10:** The gene expression under treatment of SWe^lot2^ related to capsule regulation. SH1000 biofilms were grown for 24 h, after which total RNA was extracted and the expression levels of genes associated with the *cap* operon, including *lux*, *kdpDE*, and *capAB*, were analyzed. The 16S rRNA gene was used as the internal reference for normalization. Relative gene expression in the SWe^lot2^‐treated group was determined in comparison with the untreated control group. Data are presented as the mean ± SD from at least three independent experiments. Statistical significance was evaluated using a two‐tailed one‐sample t‐test against a theoretical mean of 1. Zhao et al. ( 2010) showed that while KdpDE positively regulates the transcription of the *cap* operon, LuxS exerts the opposite effect by repressing it. However, treatment with SWe reduced the *cap* operon expression in consistence with the RNAseq result (Fig S11).
**Figure S11:** DEGs in the SWe‐treated cells: polysaccharide‐ and lysis‐related genes. Synthesis pathways of the capsule and PIA from UDP‐GlcNAc are shown top. The UDP‐N‐acetyl‐glucosamine (UDP‐N‐GlcNAc) is the building material for a series of cell surface components such as PIA, capsule, cell wall, and teichoic acid. Values of log_2_(Fold Change) relative to untreated control at the same time point are shown. SAURSH1000_RS12995 gene encodes polysaccharide biosynthesis tyrosine autokinase that is involved in the capsule assembly (RefSeq NZ_CP059180.1). C55P: undecaprenyl‐phosphate, GlcNAc: N‐acetyl‐glucosamine, FucNAc: N‐acetyl‐fucosamine, ManNAcA: N‐acetyl‐mannosaminuronic acid.
**Figure S12:** SWe reduced fibronectin binding capability. SH1000 was statically grown for 4 h in TSB with or without SWe^lot2^, and submitted to fibronectin binding assay as described in the Methods. DMSO is the solvent control. Equal cell quantities adjusted by OD were used as the input, except ‘untreated (half input)’. Means and SD of the relative values of ‘untreated’ from two independent experiments are shown. Fibronectin binding of the SWe treated cells were under the detection limit of this assay (zero in the graph).
**Figure S13:** Metabolic gene expression affected by SWe. DEGs related to the catabolism and respiration are shown. The genes coding for fatty acid β‐oxidation enzymes (*fadXDEBA*) were increased by SWe at 9 h and 24 h. These enzymes are responsible for the supplementation of acetyl‐CoA, which enters the TCA cycle. The gene coding for SdaAA and SdaAB enzyme converting serine to pyruvate were upregulated in 3 h and 24 h. In terms of electron transport, the expression of cytochrome bd oxidase (*cydAB*) was transiently increased at 3 h, and the terminal oxidases *qoxCD*, the Na + /H+ antiporter (*mnhE1*, *mnhG1*) and ATP synthase (*atpCD*) increased at 24 h. These results suggest that cells treated with SWe would have an altered respiration status, which might lead to a change in reactive oxygen species (ROS) level. Indeed, we observed the increase of ROS by SWe at 9 h (**Fig S10**).
**Figure S14:** Intracellular ROS, but not NO, is increased by SWe. After growing biofilm at indicated times, cells in planktonic and biofilm were collected together as a mixture and stained with CellROX™ (red) and DAF‐FM (Green) for detecting the intracellular ROS and NO, respectively. (A) Representative microscopic images. (B) Quantities of ROS measured by CellROX™ and (C) NO detected by DAF‐FM at 9 h and 24 h of total biomass. All data represent the average from 3 independent experiments; error bars indicate the SD. * p ≤ *0.05*. One‐way ANOVA & Dunnett's multiple comparison test with the untreated group.
**Figure S15:** Anti‐biofilm activity of SWe^lot2^ does not depend on *agr*. The appropriate concentration to observe the antibiofilm activity of SWe^lot2^ was 450 µg/mL for SH1000 and 200 µg/mL for SH1000Δagr. Higher concentration of SWe^lot2^ (e.g. 450 µg/mL) had a growth‐inhibiting effect on SH1000Δagr and not shown here. Concentrations of DMSO solvent controls are equivalent to the tested SWe^lot2^. Biofilm was formed in a 24‐well plate. SWe^lot2^ reduced the biofilm CFU (filled bars), while hardly affecting the planktonic CFU (blank bars), suggesting that anti‐biofilm activity does not rely on the presence of the Agr system. Two‐way ANOVA & Dunnett's multiple comparison test with the untreated group for each strain (***: *p* ≤ *0.0001*).
**Figure S16:** The antibiofilm activity of SWe^lot2^ on SH1000 and SA113. Cells were cultured in TSB for 24 h. Biofilm and planktonic (including washed) fractions were quantified as colony‐forming units (CFU). Data are presented as the mean ± SD of at least two independent experiments. * p ≤ 0.05 indicate significant differences compared with the untreated group. # p ≤ 0.05 indicates a significant difference between planktonic cells and the corresponding untreated control. Statistical analyses were performed using two‐way ANOVA followed by Dunnett's multiple‐comparison test against the untreated control group.
**Figure S17:** DEGs in the SWe‐treated cells: SigB‐related genes. SigB activity is post‐translationally regulated by anti‐sigma factor RsbW, and upstream regulators RsbV and RsbU. The mRNA levels of these genes were not affected by SWe. However, the *asp23* gene expression was reduced by SWe, suggesting that SigB activity is reduced by SWe. *crtMN* genes are responsible for carotenoid synthesis (see Discussion).

Supporting File 1

**Supplementary data.** Movie of biofilm status after 24 h with or without 250 µg/mL SWe or DMSO solvent control

## Data Availability

The datasets generated during the current study are available in the Zendo repository. *Links to access the data in Zendo*
1.
**Raw and processed data:**
https://zenodo.org/uploads/17529751?token=eyJhbGciOiJIUzUxMiIsImlhdCI6MTc4MjIwMDcxNiwiZXhwIjoxNzk4Njc1MTk5fQ. eyJpZCI6IjZkOGY1MDdjLTVkOTItNDJiMi1hNDJjLWMwODI1NzE3MWE2MSIsImRhdGEiOnt9LCJyYW5kb20iOiIwZGFiYThmMzFhZjE0YWVmODQxMzNlNWQ3NWM1YTdlOCJ9. Ic2s0p6Kedq2AyWjq5yr0XhlqQyCj8LMw7PbXtoGfaurMAXmTa2Fdir84J9DPh7cQ_rgNLpflof‐2rs5n2Rp4g ‐ DOI : 10.5281/zenodo.17529751 2.
**Figures for analysis**
https://zenodo.org/uploads/17529886?token=eyJhbGciOiJIUzUxMiJ9. eyJpZCI6IjdmMDkzZjUyLWNmZjMtNDY4MC05ODdjLWYzMDBmZDExYTdlMyIsImRhdGEiOnt9LCJyYW5kb20iOiIxN2E5OGYxOTBkNWIyZGIyYzIzNGFmODI1M2E2YjJhYiJ9. ogAv7cRbtKBnPoOV0yfsbkmtwLbjMpsztycpw7ThRuZATs72UxNCymbVjb9yKb7WXlr2E5M9eC4lDiWzBhjQEg ‐ DOI : 10.5281/zenodo.17529886 3.
**RNA‐seq data**
https://zenodo.org/uploads/17451855?token=eyJhbGciOiJIUzUxMiIsImlhdCI6MTc4MjIwMTI5OCwiZXhwIjoxNzk4Njc1MTk5fQ. eyJpZCI6ImI0NDBlYjA4LWY0MmYtNGNhNi1iZTRmLTJlYTA1ZjE0MDI2MSIsImRhdGEiOnt9LCJyYW5kb20iOiI1YjMwZGQzYzUyNTk1NGQ4MzI1OTgyNDA4ZjA4NzI0YiJ9. dyKGUm3EezFLUauaQ7ZZon7aAKLxhvNiSV87DVUBDXvc4cJ1ghYgpFxr3tkBQ0P9iJVTcHG7YYdFt3d‐WgW1fA ‐ DOI : 10.5281/zenodo.17451855. 4.
**Video of biofilm**
https://zenodo.org/uploads/20809578?token=eyJhbGciOiJIUzUxMiIsImlhdCI6MTc4MjIwMTY2NiwiZXhwIjoxNzk4Njc1MTk5fQ. eyJpZCI6ImIzOGNkNDhhLTQ2YmYtNDM4Ny04OGE0LTZjZmM5NTA5ZmI5NiIsImRhdGEiOnt9LCJyYW5kb20iOiJkOWIwYTM3OGU3OTQ4ZTJmYzM1MGYxNzc5YjQzYTFkMyJ9. jb7q82X0S1wefGgqGIc3FqR0o0kmuDlyfpunCEOYHKziIcxG7_1YTbqlnA00nTVc4VyCYV0mOo_ssyB5BWXSCg ‐ DOI : 10.5281/zenodo.20809578.

## References

[mbo370380-bib-0001] Alenazy, R. 2023. “Antimicrobial Activities and Biofilm Inhibition Properties of *Trigonella foenumgraecum* Methanol Extracts Against Multidrug‐Resistant *Staphylococcus aureus* and *Escherichia coli* .” Life (Basel, Switzerland) 13, no. 3: 703. 10.3390/LIFE13030703. 36983858 PMC10053055

[mbo370380-bib-0002] Biswas, R. , R. E. Martinez , N. Göhring , et al. 2012. “Proton‐Binding Capacity of *Staphylococcus aureus* Wall Teichoic Acid and Its Role in Controlling Autolysin Activity.” PLoS One 7, no. 7: e41415. 10.1371/JOURNAL.PONE.0041415. 22911791 PMC3402425

[mbo370380-bib-0003] Biswas, R. , L. Voggu , U. K. Simon , P. Hentschel , G. Thumm , and F. GÃ¶tz . 2006. “Activity of the Major Staphylococcal Autolysin Atl.” FEMS Microbiology Letters 259, no. 2: 260–268. 10.1111/J.1574-6968.2006.00281.X. 16734789

[mbo370380-bib-0004] Boles, B. R. , and A. R. Horswill . 2008. “Agr‐Mediated Dispersal of *Staphylococcus aureus* Biofilms.” PLoS Pathogens 4, no. 4: e1000052. 10.1371/journal.ppat.1000052. 18437240 PMC2329812

[mbo370380-bib-0005] Bæk, K. T. , D. Frees , A. Renzoni , et al. 2013. “Genetic Variation in the 8325 Strain Lineage Revealed by Whole‐Genome Sequencing.” PLoS One 8, no. 9: e77112. 10.1371/journal.pone.0077122. 24098817 PMC3786944

[mbo370380-bib-0006] Campoccia, D. , L. Montanaro , and C. R. Arciola . 2021. “Extracellular DNA (eDNA). A Major Ubiquitous Element of the Bacterial Biofilm Architecture.” International Journal of Molecular Sciences 22, no. 16: 9100. 10.3390/IJMS22169100. 34445806 PMC8396552

[mbo370380-bib-0007] CDC . 1999. “Four Pediatric Deaths From Community‐Acquired Methicillin‐Resistant Staphylococcus aureus—Minnesota and North Dakota, 1997‐1999.” Journal of the American Medical Association (Chicago, IL) 282, no. 12: 1123–1125. https://www.ncbi.nlm.nih.gov/pubmed/10501104. 10501104

[mbo370380-bib-0008] Chen, M. , Y. Duan , W. Yang , et al. 2025. “Role of LuxS/AI‐2 Quorum Sensing in *Staphylococcus aureus* Biofilm Formation, Intestinal Damage and Pathogenesis in Loach (*Misgurnus anguillicaudatus*).” Fish & Shellfish Immunology 167: 110691. 10.1016/j.fsi.2025.110691. 40882710

[mbo370380-bib-0009] Chen, Y. , A. T. Lun , and G. K. Smyth . 2014. “Differential Expression Analysis of Complex RNA‐Seq Experiments Using edgeR.” In Statistical Analysis of Next Generation Sequencing Data (1st ed., 51–74. Springer International Publishing: Imprint: Springer. 10.1007/978-3-319-07212-8_3.

[mbo370380-bib-0010] Chiba, A. , M. Seki , Y. Suzuki , Y. Kinjo , Y. Mizunoe , and S. Sugimoto . 2022. “ *Staphylococcus aureus* Utilizes Environmental RNA as a Building Material in Specific Polysaccharide‐Dependent Biofilms.” NPJ Biofilms and Microbiomes 8, no. 1: 17. 10.1038/s41522-022-00278-z. 35379830 PMC8980062

[mbo370380-bib-0011] Chiba, A. , S. Sugimoto , F. Sato , S. Hori , and Y. Mizunoe . 2015. “A Refined Technique for Extraction of Extracellular Matrices From Bacterial Biofilms and Its Applicability.” Microbial Biotechnology 8, no. 3: 392–403. 10.1111/1751-7915.12155. 25154775 PMC4408173

[mbo370380-bib-0012] Clauditz, A. , A. Resch , K. P. Wieland , A. Peschel , and F. Götz . 2006. “Staphyloxanthin Plays a Role in the Fitness of *Staphylococcus aureus* and Its Ability to Cope With Oxidative Stress.” Infection and Immunity 74, no. 8: 4950–4953. 10.1128/IAI.00204-06/ASSET/0B500F87-F8F6-43EC-8C7E-02D1C33F109E/ASSETS/GRAPHIC/ZII0080661110005.JPEG. 16861688 PMC1539600

[mbo370380-bib-0013] CLSI M26‐A . n.d.‐ Methods for Determining Bactericidal Activity of Antimicrobial Agents; Approved Guideline. In.

[mbo370380-bib-0014] Dengler, V. , L. Foulston , A. S. DeFrancesco , and R. Losick . 2015. “An Electrostatic Net Model for the Role of Extracellular DNA in Biofilm Formation by *Staphylococcus aureus* .” Journal of Bacteriology 197, no. 24: 3779–3787. 10.1128/JB.00726-15. 26416831 PMC4652055

[mbo370380-bib-0015] Eddenden, A. , and M. Nitz . 2022. “Applications of an Inactive Dispersin B Probe to Monitor Biofilm Polysaccharide Production.” Method Enzymol 665: 209–231. 10.1016/bs.mie.2021.11.006. 35379435

[mbo370380-bib-0016] Ford, C. A. , I. M. Hurford , and J. E. Cassat . 2020. “Antivirulence Strategies for the Treatment of *Staphylococcus aureus* Infections: A Mini Review.” Frontiers in Microbiology 11: 632706. 10.3389/FMICB.2020.632706. 33519793 PMC7840885

[mbo370380-bib-0017] Gagnon, E. , A. Bruneau , C. E. Hughes , L. de Queiroz , and G. P. Lewis . 2016. “A New Generic System for the Pantropical *Caesalpinia* Group (*Leguminosae*).” PhytoKeys 71: 1–160. 10.3897/phytokeys.71.9203. PMC5558824 28814915

[mbo370380-bib-0018] Gomes, F. , N. Martins , I. C. F. R. Ferreira , and M. Henriques . 2019. “Anti‐Biofilm Activity of Hydromethanolic Plant Extracts Against *Staphylococcus aureus* Isolates From Bovine Mastitis.” Heliyon 5, no. 5: e01728. 10.1016/J.HELIYON.2019.E01728. 31193536 PMC6535580

[mbo370380-bib-0019] Gor, V. , A. J. Takemura , M. Nishitani , et al. 2019. “Finding of Agr Phase Variants in *Staphylococcus aureus* .” mBio 10, no. 4: e00796‐19. 10.1128/MBIO.00796-19. 31387900 PMC6686034

[mbo370380-bib-0020] Graf, A. C. , A. Leonard , M. Schäuble , et al. 2019. “Virulence Factors Produced by *Staphylococcus aureus* Biofilms Have a Moonlighting Function Contributing to Biofilm Integrity.” Molecular & Cellular Proteomics: MCP 18, no. 6: 1036–1053. 10.1074/MCP.RA118.001120. 30850421 PMC6553939

[mbo370380-bib-0021] Grando, K. , L. K. Nicastro , S. A. Tursi , et al. 2022. “Phenol‐Soluble Modulins From Staphylococcus aureus Biofilms Form Complexes With DNA to Drive Autoimmunity.” Frontiers in Cellular and Infection Microbiology 12: 884065. 10.3389/fcimb.2022.884065. 35646719 PMC9131096

[mbo370380-bib-0022] Gries, C. M. , T. Biddle , J. L. Bose , T. Kielian , and D. D. Lo . 2020. “ *Staphylococcus aureus* Fibronectin Binding Protein A Mediates Biofilm Development and Infection.” Infection and Immunity 88, no. 5: e00859‐19. 10.1128/IAI.00859-19. 32041788 PMC7171244

[mbo370380-bib-0023] Guo, Y. , G. Song , M. Sun , J. Wang , and Y. Wang . 2020. “Prevalence and Therapies of Antibiotic‐Resistance in *Staphylococcus aureus* .” Frontiers in Cellular and Infection Microbiology 10: 107. 10.3389/FCIMB.2020.00107. 32257966 PMC7089872

[mbo370380-bib-0024] Horsburgh, M. J. , J. L. Aish , I. J. White , L. Shaw , J. K. Lithgow , and S. J. Foster . 2002. “σB Modulates Virulence Determinant Expression and Stress Resistance: Characterization of a Functional *rsb*U Strain Derived From *Staphylococcus aureus* 8325‐4.” Journal of Bacteriology 184, no. 19: 5457–5467. 10.1128/JB.184.19.5457-5467.2002. 12218034 PMC135357

[mbo370380-bib-0025] Howard, M. K. , K. R. Miller , B. S. Sohn , J. J. Ryan , A. Xu , and M. E. Jackrel . 2023. “Probing the Drivers of Staphylococcus Aureus Biofilm Protein Amyloidogenesis and Disrupting Biofilms With Engineered Protein Disaggregases.” mBio 14, no. 4: 0058723. 10.1128/mbio.00587-23. PMC10470818 37195208

[mbo370380-bib-0026] Humphreys, H. 2012. “ *Staphylococcus aureus*: The Enduring Pathogen in Surgery.” Surgeon 10, no. 6: 357–360. 10.1016/J.SURGE.2012.05.003. 23079115

[mbo370380-bib-0027] Iordanescu, S. , and M. Surdeanu . 1976. “Two Restriction and Modification Systems in *Staphylococcus aureus* NCTC8325.” Journal of General Microbiology 96, no. 2: 277–281. 10.1099/00221287-96-2-277. 136497

[mbo370380-bib-0028] Izano, E. A. , M. A. Amarante , W. B. Kher , and J. B. Kaplan . 2008. “Differential Roles of Poly‐N‐Acetylglucosamine Surface Polysaccharide and Extracellular DNA in *Staphylococcus aureus* and *Staphylococcus epidermidis* Biofilms.” Applied and Environmental Microbiology 74, no. 2: 470–476. 10.1128/AEM.02073-07. 18039822 PMC2223269

[mbo370380-bib-0029] Jacqueline, C. , and J. Caillon . 2014. “Impact of Bacterial Biofilm on the Treatment of Prosthetic Joint Infections.” The Journal of Antimicrobial Chemotherapy 69 Suppl 1, no. SUPPL1: i37‐i40. 10.1093/JAC/DKU254. 25135088

[mbo370380-bib-0030] Jiang, F. , Y. Chen , J. Yu , et al. 2023. “Repurposed Fenoprofen Targeting SaeR Attenuates *Staphylococcus aureus* Virulence in Implant‐Associated Infections.” ACS Central Science 9, no. 7: 1354–1373. 10.1021/ACSCENTSCI.3C00499/SUPPL_FILE/OC3C00499_SI_002.XLSX. 37521790 PMC10375895

[mbo370380-bib-0031] Jordan, S. C. , P. R. Hall , and S. M. Daly . 2022. “Nonconformity of Biofilm Formation *In Vivo* and *In Vitro* Based on Accessory Gene Regulator Status.” Scientific Reports 12: 1251. 10.1038/s41598-022-05382-w. 35075262 PMC8786897

[mbo370380-bib-0032] Kaito, C. , and K. Sekimizu . 2007. “Colony Spreading in *Staphylococcus aureus* .” Journal of Bacteriology 189, no. 6: 2553–2557. 10.1128/JB.01635-06. 17194792 PMC1899384

[mbo370380-bib-0033] Kavanaugh, J. S. , C. E. Flack , J. Lister , et al. 2019. “Identification of Extracellular DNA‐Binding Proteins in the Biofilm Matrix.” mBio 10, no. 3: e01137‐19. https://journals.asm.org/doi/10.1128/mbio.01137-19. 31239382 10.1128/mBio.01137-19 PMC6593408

[mbo370380-bib-0034] Kim, T. , H. D. Seo , L. Hennighausen , D. Lee , and K. Kang . 2018. “Octopus‐Toolkit: A Workflow to Automate Mining of Public Epigenomic and Transcriptomic Next‐Generation Sequencing Data.” Nucleic Acids Research 46, no. 9: e53. 10.1093/nar/gky083. 29420797 PMC5961211

[mbo370380-bib-0035] Kizaki, H. , Y. Omae , F. Tabuchi , Y. Saito , K. Sekimizu , and C. Kaito . 2016. “Cell‐Surface Phenol Soluble Modulins Regulate *Staphylococcus aureus* Colony Spreading.” PLoS One 11, no. 10: e0164523. 10.1371/JOURNAL.PONE.0164523. 27723838 PMC5056675

[mbo370380-bib-0036] Kullik, I. , P. Giachino , and T. Fuchs . 1998. “Deletion of the Alternative Sigma Factor σ(B) in *Staphylococcus aureus* Reveals Its Function as a Global Regulator of Virulence Genes.” Journal of Bacteriology 180, no. 18: 4814–4820. 10.1128/JB.180.18.4814-4820.1998/ASSET/328A9147-5F90-4DC9-8702-FFFDAD4877F9/ASSETS/GRAPHIC/JB1880515005.JPEG. 9733682 PMC107504

[mbo370380-bib-0037] Lan, L. , A. Cheng , P. M. Dunman , D. Missiakas , and C. He . 2010. “Golden Pigment Production and Virulence Gene Expression Are Affected by Metabolisms in *Staphylococcus aureus* .” Journal of Bacteriology 192, no. 12: 3068–3077. 10.1128/JB.00928-09. 20400547 PMC2901709

[mbo370380-bib-0038] Le, K. Y. , and M. Otto . 2015. “Quorum‐Sensing Regulation in Staphylococci‐An Overview.” Frontiers in Microbiology 6, no. OCT: 167362. 10.3389/FMICB.2015.01174/BIBTEX. PMC4621875 26579084

[mbo370380-bib-0039] Lee, J. H. , Y. G. Kim , J. G. Park , and J. Lee . 2017. “Supercritical Fluid Extracts of *Moringa oleifera* and Their Unsaturated Fatty Acid Components Inhibit Biofilm Formation by *Staphylococcus aureus* .” Food Control 80: 74–82. 10.1016/J.FOODCONT.2017.04.035.

[mbo370380-bib-0040] Liao, Y. , G. K. Smyth , and W. Shi . 2014. “Featurecounts: An Efficient General Purpose Program for Assigning Sequence Reads to Genomic Features.” Bioinformatics 30, no. 7: 923–930. 10.1093/bioinformatics/btt656. 24227677

[mbo370380-bib-0041] Liu, Q. , W. S. Yeo , and T. Bae . 2016. “The Saers Two‐Component System of *Staphylococcus aureus* .” Genes 7, no. 10: 81. 10.3390/GENES7100081. 27706107 PMC5083920

[mbo370380-bib-0042] Liu, S. , F. Le Mauff , D. C. Sheppard , and S. Zhang . 2022. “Filamentous Fungal Biofilms: Conserved and Unique Aspects of Extracellular Matrix Composition, Mechanisms of Drug Resistance and Regulatory Networks in *Aspergillus fumigatus* .” NPJ Biofilms and Microbiomes 8, no. 1: 83. 10.1038/s41522-022-00347-3. 36261442 PMC9581972

[mbo370380-bib-0043] Mahdally, N. H. , R. F. George , M. T. Kashef , M. Al‐Ghobashy , F. E. Murad , and A. S. Attia . 2021. “Staquorsin: A Novel *Staphylococcus Aureus* Agr‐Mediated Quorum Sensing Inhibitor Impairing Virulence *IN Vivo* Without Notable Resistance Development.” Frontiers in Microbiology 12: 700494. 10.3389/fmicb.2021.700494. 34290689 PMC8287904

[mbo370380-bib-0044] Mann, E. E. , K. C. Rice , B. R. Boles , et al. 2009. “Modulation of eDNA Release and Degradation Affects *Staphylococcus aureus* Biofilm Maturation.” PLoS One 4, no. 6: e5822. 10.1371/JOURNAL.PONE.0005822. 19513119 PMC2688759

[mbo370380-bib-0045] Marroquin, S. , B. Gimza , B. Tomlinson , et al. 2019. “Mroq Is a Novel Abi‐Domain Protein That Influences Virulence Gene Expression in *Staphylococcus aureus* via Modulation of *Agr* Activity.” Infection and Immunity 87, no. 5: e00002‐19. https://journals.asm.org/doi/10.1128/iai.00002-19. 30833335 10.1128/IAI.00002-19 PMC6479029

[mbo370380-bib-0046] Mashruwala, A. A. , C. M. Gries , T. D. Scherr , T. Kielian , and J. M. Boyd . 2017. “SaeRS Is Responsive to Cellular Respiratory Status and Regulates Fermentative Biofilm Formation in *Staphylococcus aureus* .” Infection and Immunity 85, no. 8: e00157‐17. 10.1128/IAI.00157-17. 28507069 PMC5520442

[mbo370380-bib-0047] McCarthy, H. , J. K. Rudkin , N. S. Black , L. Gallagher , E. O'Neill , and J. P. O'Gara . 2015. “Methicillin Resistance and the Biofilm Phenotype in *Staphylococcus aureus* .” Frontiers in Cellular and Infection Microbiology 5: 1. 10.3389/fcimb.2015.00001. 25674541 PMC4309206

[mbo370380-bib-0048] Moormeier, D. E. , and K. W. Bayles . 2017. “ *Staphylococcus aureus* Biofilm: A Complex Developmental Organism.” Molecular Microbiology 104, no. 3: 365–376. 10.1111/MMI.13634. 28142193 PMC5397344

[mbo370380-bib-0049] Moormeier, D. E. , J. L. Bose , A. R. Horswill , and K. W. Bayles . 2014. “Temporal and Stochastic Control of *Staphylococcus aureus* Biofilm Development.” mBio 5, no. 5: e01341‐14. 10.1128/MBIO.01341-14. 25316695 PMC4205790

[mbo370380-bib-0050] Morales‐Laverde, L. , M. Echeverz , M. Trobos , C. Solano , and I. Lasa . 2022. “Experimental Polymorphism Survey in Intergenic Regions of the *ica*ADBCR Locus in *Staphylococcus aureus* Isolates From Periprosthetic Joint Infections.” Microorganisms 10, no. 3: 600. 10.3390/MICROORGANISMS10030600. 35336176 PMC8955882

[mbo370380-bib-0051] Morikawa, K. , A. Maruyama , Y. Inose , M. Higashide , H. Hayashi , and T. Ohta . 2001. “Overexpression of Sigma Factor, ςB, Urges Staphylococcus Aureus to Thicken the Cell Wall and to Resist Β‐Lactams.” Biochemical and Biophysical Research Communications 288, no. 2: 385–389. 10.1006/bbrc.2001.5774. 11606054

[mbo370380-bib-0052] Mrak, L. N. , A. K. Zielinska , K. E. Beenken , et al. 2012. “ *sae*RS and *sar*A Act Synergistically to Repress Protease Production and Promote Biofilm Formation in *Staphylococcus aureus* .” PLoS One 7, no. 6: e38453. 10.1371/JOURNAL.PONE.0038453. 22685571 PMC3369899

[mbo370380-bib-0053] Muhs, A. , J. T. Lyles , C. P. Parlet , et al. 2017. “Virulence Inhibitors From Brazilian Peppertree Block Quorum Sensing and Abate Dermonecrosis in Skin Infection Models.” Scientific Reports 7, no. 1: 42275. 10.1038/srep42275. 28186134 PMC5301492

[mbo370380-bib-0054] Murray, C. J. L. , K. S. Ikuta , F. Sharara , et al. 2022. “Global Burden of Bacterial Antimicrobial Resistance in 2019: A Systematic Analysis.” Lancet 399, no. 10325: 629–655. 10.1016/S0140-6736(21)02724-0. 35065702 PMC8841637

[mbo370380-bib-0055] Ngernnak, C. , P. Panyajai , S. Anuchapreeda , W. Wongkham , and A. Saiai . 2018. “Phytochemical AND Cytotoxic Investigations of the Heartwood OF *Caesalpinia Sappan* LINN.” Asian Journal of Pharmaceutical and Clinical Research 11, no. 2: 336–339. 10.22159/AJPCR.2018.V11I2.22903.

[mbo370380-bib-0056] Nguyen, H. T. T. , T. H. Nguyen , and M. Otto . 2020. “The Staphylococcal Exopolysaccharide Pia – Biosynthesis and Role in Biofilm Formation, Colonization, and Infection.” Computational and Structural Biotechnology Journal 18: 3324–3334. 10.1016/J.CSBJ.2020.10.027. 33240473 PMC7674160

[mbo370380-bib-0057] Nishitani, K. , W. Sutipornpalangkul , K. L. De Mesy Bentley , et al. 2015. “Quantifying the Natural History of Biofilm Formation In Vivo During the Establishment of Chronic Implant‐Associated *Staphylococcus aureus* Osteomyelitis in Mice to Identify Critical Pathogen and Host Factors.” Journal of Orthopaedic Research 33, no. 9: 1311–1319. 10.1002/JOR.22907. 25820925 PMC4529770

[mbo370380-bib-0058] Olson, M. E. , T. K. Nygaard , L. Ackermann , et al. 2013. “ *Staphylococcus aureus* nuclease Is an SaeRS‐Dependent Virulence Factor.” Infection and Immunity 81, no. 4: 1316–1324. 10.1128/IAI.01242-12. 23381999 PMC3639593

[mbo370380-bib-0059] O'Neill, E. , C. Pozzi , P. Houston , et al. 2007. “Association Between Methicillin Susceptibility and Biofilm Regulation in *Staphylococcus aureus* Isolates From Device‐Related Infections.” Journal of Clinical Microbiology 45, no. 5: 1379–1388. 10.1128/JCM.02280-06. 17329452 PMC1865887

[mbo370380-bib-0060] Paharik, A. E. , and A. R. Horswill . 2016. “The Staphylococcal Biofilm: Adhesins, Regulation, and Host Response.” Microbiology Spectrum 4, no. 2: vmbf‐0022‐2015. 10.1128/microbiolspec.VMBF-0022-2015. PMC4887152 27227309

[mbo370380-bib-0061] Peng, D. , A. Chen , B. Shi , et al. 2018. “Preliminary Study on the Effect of Brazilin on Biofilms of *Staphylococcus aureus* .” Experimental and Therapeutic Medicine 16, no. 3: 2108–2118. 10.3892/ETM.2018.6403. 30186447 PMC6122259

[mbo370380-bib-0062] Periasamy, S. , H. S. Joo , A. C. Duong , et al. 2012. “How *Staphylococcus Aureus* Biofilms Develop Their Characteristic Structure.” Proceedings of the National Academy of Sciences 109, no. 4: 1281–1286. 10.1073/pnas.1115006109. PMC3268330 22232686

[mbo370380-bib-0063] Rice, K. C. , E. E. Mann , J. L. Endres , et al. 2007. “The *Cid*A Murein Hydrolase Regulator Contributes to Dna Release and Biofilm Development in *Staphylococcus aureus* .” Proceedings of the National Academy of Sciences 104, no. 19: 8113–8118. 10.1073/PNAS.0610226104. PMC1876580 17452642

[mbo370380-bib-0064] Schindelin, J. , I. Arganda‐Carreras , E. Frise , et al. 2012. “Fiji: An Open‐Source Platform for Biological‐Image Analysis.” Nature Methods 9, no. 7: 676–682. 10.1038/Nmeth.2019. 22743772 PMC3855844

[mbo370380-bib-0065] Schurig‐Briccio, L. A. , P. K. Parraga Solorzano , A. M. Lencina , et al. 2020. “Role of Respiratory NADH Oxidation in the Regulation of *Staphylococcus aureus* Virulence.” The EMBO Reports 21, no. 5: e45832. 10.15252/embr.201845832. 32202364 PMC7202225

[mbo370380-bib-0066] Schwartz, K. , A. K. Syed , R. E. Stephenson , A. H. Rickard , and B. R. Boles . 2012. “Functional Amyloids Composed of Phenol Soluble Modulins Stabilize *Staphylococcus aureus* Biofilms.” PLoS Pathogens 8, no. 6: e1002744. 10.1371/JOURNAL.PPAT.1002744. 22685403 PMC3369951

[mbo370380-bib-0067] Seidl, K. , C. Goerke , C. Wolz , D. Mack , B. Berger‐Bächi , and M. Bischoff . 2008. “ *Staphylococcus aureus* CcpA Affects Biofilm Formation.” Infection and Immunity 76, no. 5: 2044–2050. 10.1128/IAI.00035-08. 18347047 PMC2346702

[mbo370380-bib-0068] Shopova, I. , S. Bruns , A. Thywissen , O. Kniemeyer , A. A. Brakhage , and F. Hillmann . 2013. “Extrinsic Extracellular DNA Leads to Biofilm Formation and Colocalizes With Matrix Polysaccharides in the Human Pathogenic Fungus *Aspergillus fumigatus* .” Frontiers in Microbiology 4, no. JUN: 141. 10.3389/FMICB.2013.00141. 23760756 PMC3674311

[mbo370380-bib-0069] Sugimoto, S. , F. Sato , R. Miyakawa , et al. 2018. “Broad Impact of Extracellular DNA on Biofilm Formation by Clinically Isolated Methicillin‐Resistant and ‐Sensitive Strains of *Staphylococcus aureus* .” Scientific Reports 8, no. 1: 2254. 10.1038/s41598-018-20485-z. 29396526 PMC5797107

[mbo370380-bib-0070] Tan, L. , Y. Huang , W. Shang , et al. 2022. “Accessory Gene Regulator (Agr) Allelic Variants in Cognate *Staphylococcus aureus* Strain Display Similar Phenotypes.” Frontiers in Microbiology 13: 700894. 10.3389/FMICB.2022.700894/BIBTEX. 35295312 PMC8919982

[mbo370380-bib-0071] Tan, X. , N. Qin , C. Wu , et al. 2015. “Transcriptome Analysis of the Biofilm Formed by Methicillin‐Susceptible *Staphylococcus aureus* .” Scientific Reports 2015 5:1 5, no. 1: 1–12. 10.1038/srep11997. PMC4493712 26149474

[mbo370380-bib-0072] Tang, H. , G. Porras , M. M. Brown , et al. 2020. “Triterpenoid Acids Isolated From Schinus Terebinthifolia Fruits Reduce *Staphylococcus aureus* Virulence and Abate Dermonecrosis.” Scientific Reports 10, no. 1: 8046. 10.1038/s41598-020-65080-3. 32415287 PMC7229044

[mbo370380-bib-0073] Tayeb‐Fligelman, E. , O. Tabachnikov , A. Moshe , et al. 2017. “The Cytotoxic *Staphylococcus aureus* PSMα3 Reveals a Cross‐α Amyloid‐Like Fibril.” Science 355, no. 6327: 831–833. 10.1126/SCIENCE.AAF4901/SUPPL_FILE/TAYEB-FLIGELMAN.SM.PDF. 28232575 PMC6372758

[mbo370380-bib-0074] Trotonda, P. , S. Tamber , G. Memmi , and A. L. Cheung . 2008. “MgrA Represses Biofilm Formation in *Staphylococcus aureus* .” Infection and Immunity 76, no. 12: 5645–5654. 10.1128/IAI.00735-08. 18852246 PMC2583562

[mbo370380-bib-0075] Tuchscherr, L. , and M. Otto . 2023. “Critical Assessment of the Prospects of Quorum‐Quenching Therapy for *Staphylococcus aureus* Infection.” International Journal of Molecular Sciences 2023, Vol. 24, Page 4025 24, no. 4: 4025. 10.3390/IJMS24044025. PMC9958572 36835436

[mbo370380-bib-0076] Vij, T. , P. P. Anil , R. Shams , et al. 2023. “A Comprehensive Review on Bioactive Compounds Found in *Caesalpinia sappan* .” Molecules 28, no. 17: 6247. 10.3390/MOLECULES28176247. 37687076 PMC10488625

[mbo370380-bib-0077] Vila, T. , E. F. Kong , A. Ibrahim , et al. 2019. “Candida Albicans Quorum‐Sensing Molecule Farnesol Modulates Staphyloxanthin Production and Activates the Thiol‐Based Oxidative‐Stress Response in Staphylococcus Aureus.” Virulence 10, no. 1: 625–642. 10.1080/21505594.2019.1635418. 31280653 PMC6629188

[mbo370380-bib-0078] Vinodhini, V. , and M. Kavitha . 2024. “Deciphering Agr Quorum Sensing in *Staphylococcus aureu*S: Insights and Therapeutic Prospects.” Molecular Biology Reports 51, no. 1: 155. 10.1007/S11033-023-08930-3. 38252331

[mbo370380-bib-0079] Vuong, C. , H. L. Saenz , F. Götz , and M. Otto . 2000. “Impact of the Agr Quorum‐Sensing System on Adherence to Polystyrene in *Staphylococcus aureus* .” The Journal of infectious diseases 182, no. 6: 1688–1693. 10.1086/317606. 11069241

[mbo370380-bib-0080] Wang, H. Y. , X. W. Hua , H. R. Jia , et al. 2016. “Universal Cell Surface Imaging for Mammalian, Fungal, and Bacterial Cells.” ACS Biomaterials Science & Engineering 2, no. 6: 987–997. 10.1021/acsbiomaterials.6b00130. 33429507

[mbo370380-bib-0081] Xue, L. , Y. Y. Chen , Z. Yan , W. Lu , D. Wan , and H. Zhu . 2019. “Staphyloxanthin: A Potential Target for Antivirulence Therapy.” Infection and Drug Resistance 12: 2151–2160. 10.2147/IDR.S193649. 31410034 PMC6647007

[mbo370380-bib-0082] Zhao, L. , T. Xue , F. Shang , H. Sun , and B. Sun . 2010. “ *Staphylococcus aureus* AI‐2 Quorum Sensing Associates With the KdpDE Two‐Component System to Regulate Capsular Polysaccharide Synthesis and Virulence.” Infection and Immunity 78, no. 8: 3506–3515. 10.1128/IAI.00131-10. 20498265 PMC2916273

